# MUC1-ARF—A Novel MUC1 Protein That Resides in the Nucleus and Is Expressed by Alternate Reading Frame Translation of MUC1 mRNA

**DOI:** 10.1371/journal.pone.0165031

**Published:** 2016-10-21

**Authors:** Michael Chalick, Oded Jacobi, Edward Pichinuk, Christian Garbar, Armand Bensussan, Alan Meeker, Ravit Ziv, Tania Zehavi, Nechama I. Smorodinsky, John Hilkens, Franz-Georg Hanisch, Daniel B. Rubinstein, Daniel H. Wreschner

**Affiliations:** 1 Department of Cell Research and Immunology, Tel Aviv University, Ramat Aviv, Israel; 2 Department of Biopathology, Institut Jean-Godinot, Reims Cedex, France; 3 INSERM U976, Hôpital Saint Louis, Paris, France; 4 The Johns Hopkins University School of Medicine, Baltimore, Maryland, United States of America; 5 Department of Pathology, Meir Medical Center, Kfar Saba, Israel; 6 Division of Molecular Genetics, The Netherlands Cancer Institute, Amsterdam, The Netherlands; 7 Institute of Biochemistry II, Medical Faculty, University of Cologne, Köln, Germany; 8 BioModifying LLC, Silver Spring, Maryland, United States of America; University of Nebraska Medical Center, UNITED STATES

## Abstract

Translation of mRNA in alternate reading frames (ARF) is a naturally occurring process heretofore underappreciated as a generator of protein diversity. The *MUC1* gene encodes MUC1-TM, a signal-transducing trans-membrane protein highly expressed in human malignancies. Here we show that an AUG codon downstream to the MUC1-TM initiation codon initiates an alternate reading frame thereby generating a novel protein, MUC1-ARF. MUC1-ARF, like its MUC1-TM 'parent’ protein, contains a tandem repeat (VNTR) domain. However, the amino acid sequence of the MUC1-ARF tandem repeat as well as N- and C- sequences flanking it differ entirely from those of MUC1-TM. In vitro protein synthesis assays and extensive immunohistochemical as well as western blot analyses with MUC1-ARF specific monoclonal antibodies confirmed MUC1-ARF expression. Rather than being expressed at the cell membrane like MUC1-TM, immunostaining showed that MUC1-ARF protein localizes mainly in the nucleus: Immunohistochemical analyses of MUC1-expressing tissues demonstrated MUC1-ARF expression in the nuclei of secretory luminal epithelial cells. MUC1-ARF expression varies in different malignancies. While the malignant epithelial cells of pancreatic cancer show limited expression, in breast cancer tissue MUC1-ARF demonstrates strong nuclear expression. Proinflammatory cytokines upregulate expression of MUC1-ARF protein and co-immunoprecipitation analyses demonstrate association of MUC1-ARF with SH3 domain-containing proteins. Mass spectrometry performed on proteins coprecipitating with MUC1-ARF demonstrated Glucose-6-phosphate 1-dehydrogenase (G6PD) and Dynamin 2 (DNM2). These studies not only reveal that the *MUC1* gene generates a previously unidentified MUC1-ARF protein, they also show that just like its ‘parent’ MUC1-TM protein, MUC1-ARF is apparently linked to signaling and malignancy, yet a definitive link to these processes and the roles it plays awaits a precise identification of its molecular functions. Comprising at least 524 amino acids, MUC1-ARF is, furthermore, the longest ARF protein heretofore described.

## Introduction

Any AUG codon within a given mRNA sequence may potentially function as an initiation site for translation, provided that it is located within an appropriate extended nucleotide sequence context that can support translational initiation. Specifically, an in-frame N-terminally extended protein can be generated by translation initiated by an in-frame AUG start codon located 5' to a downstream start codon. Accordingly, deep proteome analyses have identified at least sixteen novel AUG start sites that give rise to N-terminally extended protein variants, in addition to four translated upstream ORFs [1]. Alternatively, start codons appearing at additional sites within the mRNA sequence can initiate mRNA translation in alternate reading frames (ARFs) yielding a peptide sequence differing entirely from the ‘parent’ protein product [2].

In viruses, utilization of alternate reading frames contributes to diversification of the protein repertoire that can be generated from the viral genome, whilst at the same time keeping it compact[3]. In contrast to viruses, in eukaryotic organisms and in humans in particular there have been relatively few definitive reports of translation in alternate reading frames yielding proteins differing from their 'parent' proteins [4–7]. The best-defined eukaryotic ARF protein studied thus far derives from the INK4-ARF locus, which generates two alternative transcripts that use different alternate frames of a constitutive exon to encode the tumor suppressor proteins p16^INK4a^ and p19^ARF^ [8]. These proteins inhibit cyclin dependent kinases (CDK4 and CDK6), thereby preventing phosphorylation and allowing the non-phosphorylated RB proteins to act as suppressors of cell growth [9]. Interestingly, the corresponding 'parent' protein p16^INK4A^ and the alternatively translated p19^ARF^ both act in shared pathways of tumor suppression.

Additional examples of well-defined ARF proteins include those derived from the mRNAs coding for the stimulatory G-protein specific to neuroendocrine cells (termed ALEX) [10, 11] and MASK-BP3 [12]. Despite their completely different amino acid sequences, functions mediated by these pairs of 'parent' and ARF proteins are intimately intertwined and also involve physical interaction between the ‘parental’ and the ARF proteins. In contrast to the limited number of well-characterized ARF proteins generated from the mammalian genome as described above, a recent publication suggests that translation in an alternate reading frame may, to the contrary, be much more prevalent than previously anticipated [2].

*MUC1*, a gene recognized for more than two decades, is clearly related to a malignant cell phenotype [13–15]. Its major protein product, the transmembrane MUC1-TM protein, is highly expressed over the entire cell surface of tumor cells from a variety of epithelial malignancies, whereas in normal epithelial cells its expression is not only considerably lower but is also restricted solely to the apical surface of epithelial cells that form luminal structures [16]. We demonstrate here for the first time that translation in an alternative reading frame of mRNA coding for MUC1-TM yields a novel protein, here designated MUC1-ARF. MUC1-ARF comprises an amino acid sequence entirely different from that of MUC1-TM and represents the largest eukaryotic protein derived from translation of an mRNA in an alternate reading frame reported to date. In contrast to localization of MUC1-TM on the cell surface, MUC1-ARF locates primarily to the cell nucleus. Luminal epithelial cells of tissues such as pancreas and kidney, which normally express significant levels of MUC1-TM also express MUC1-ARF, while tissues that express low levels of MUC1-TM such as breast luminal-forming epithelial cells express very low levels of MUC1-ARF. In contrast, many breast cancers express MUC1-TM at high levels and of these, a discrete subset shows high MUC1-ARF expression. We show here that functionally, MUC1-ARF interacts with signaling proteins, including those comprising SH3 domains and mass spectrometry showed coprecipitation of MUC1-ARF with G6PD and Dynamin 2. While suggestive of a link between MUC1-ARF, signaling, and malignancy, definitive demonstration of such a link awaits identification of MUC1-ARF’s precise function. In summary, the studies presented here demonstrate that the *MUC1* gene generates the novel MUC1-ARF protein by translation of MUC1 mRNA in an alternate reading frame. Moreover, MUC1-ARF is, to our knowledge, the longest ARF protein heretofore described.

## Materials and Methods

### Cell lines and cell culture

DA3-TM mouse mammary tumor cells transfected with, and expressing cDNA coding for full-length MUC1-TM [17], DA3-PAR non-transfected parental DA3 cells [17], human breast carcinoma cell lines T47D and ZR75 [17], and human pancreatic carcinoma cell line Colo357 [17] were grown in Dulbecco's Modified Eagle's Medium (DMEM), RPMI and DMEM: F12 (1:1) culture media. Human cell lines were authenticated by Short Tandem Repeat (STR) analysis (PowerPlexW 1.2 System, Promega, WI, US) and the STR profiles were matched to the German Collection of Microorganisms and Cell Cultures (DSMZ) database. Prior to treatment with cytokine, Colo357 cells were incubated in serum-free medium for 24 hrs. Cells were then treated at the following concentrations: IL-1beta (20ng/ml), IL-6 (40 ng/ml), TNF-alpha (20 ng/ml), or Interferon-gamma (10 ng/ml).

### Whole cell lysates

Cells were washed with PBS, scraped from culture flasks and pelleted. Two volumes of cold lysis buffer [50mM Tris, 100mM NaCl, 1% Triton X-100 + protease inhibitor cocktail (Sigma)] were added to the cell pellet followed by incubation on ice for 30 minutes and centrifuged for 20 minutes at 12000 RPM. Concentration of protein samples were determined by BCA assay.

### Nuclear and cytoplasmic lysates

Cell pellets were resuspended in extraction buffer [0.3M sucrose, 50mM Tris pH7.4 +protease inhibitor cocktail (Sigma)] and disrupted by 10 freeze and thaw cycles on dry ice. This was followed by centrifugation 20 minutes at 14000 rpm, and the supernatant collected as the cytoplasmic protein solution. The pellet was suspended in nuclear extraction buffer ([50mM Tris, 100 mM NaCl, 1% Triton X-100 + protease inhibitor cocktail (Sigma)] and sonicated for 30 seconds with a 1 minute rest 10 times at power setting 5. This was followed by centrifugation at 14,000 rpm for 20 minutes at 4°C, with the supernatant representing the nuclear protein solution.

### Immunization of mice and generation of hybridomas

Mice were immunized with MUC1-ARF peptide conjugated to KLH. The MUC1-ARF peptide used for immunization comprised 1.3 repeats of the MUC1-ARF repeat unit, PQPTVSPRPRTPGRPRAPPP-PQPTVS- (one 20 amino acid long repeat is underlined and six amino acids of the following repeat is double-underlined). Use of animals was performed under the supervision of Tel Aviv University Institutional Animal Care and Use Committee (TAU-IACUC), License number L-08-026. Animal welfare and steps taken to ameliorate suffering including methods of sacrifice were all performed in accordance with regulations stipulated by TAU-IACUC. Following immunization, samples of polyclonal sera were taken and antibody titers assessed using an ELISA assay wherein BSA-MUC1-ARF peptide was coated onto the well surface of 96 well plates. Spleen cells from immunized mice were fused with mouse myeloma NS0 and selected in HAT medium according to the standard protocol.

### Three-tiered screening for selection of anti-MUC1-ARF monoclonal antibodies

The primary screen of the hybridomas was performed by assessing antibody present in hybridoma supernatants binding to MUC1-ARF peptide. Positive hybridomas were then submitted to a secondary screen wherein hybridoma supernatants were assessed for binding by western blot analysis using lysates from MUC1 transfected cells. Hybridomas that were positive on both the primary and secondary screens were finally assessed using immunofluorescence assay on DA3 mouse cells that do not express human MUC1 (as negative control), and on stable DA3 transfectants expressing human MUC1. Hybridomas secreting antibody that scored positive in all three screens were subsequently cloned by repeated limiting dilution until a stable clone was obtained.

*Immunofluorescence—*Cells (50,000 cells per well) were seeded on glass cover-slips in 24-well culture plates. The next day, cells were rinsed with ice-cold PBS and fixed with 100% methanol for 5 min at -20°C followed by permeabilization with methanol/acetone (1:1). The cells were subjected to immunofluorescence staining with primary antibody for 1h at room temperature, then washed and incubated with fluorescently-labeled secondary antibody at room temperature for an additional 1 h. The cells were examined by fluorescence confocal microscopy.

### Immunohistochemistry

Tissue microarrays were purchased from US Biomax, Inc. URL- http://biomax.us. Tissue sections (paraffin embedded, 5 microns in thickness) were deparaffinized followed by rehydration. Automated Immunological stains were performed with the Dako Autostainer Link 48 (Dako) according to the manufacturer’s instructions. Antigen retrieval was done using citrate buffer for 30 minutes at room temperature, or at alkaline pH (Tris-EDTA, pH9.0) for the anti-MUC1-ARF antibodies. Endogenous peroxidase activity was blocked with EnVision Flex Peroxidase Blocking Reagent (Dako) for 30 minutes, followed by incubation with primary antibody (5microgram/ml) for 120 minutes. The immunological reaction was revealed by means of polymer dextran coupled with peroxidase molecule and secondary antibodies for 15 minutes (EnVision-Flex /HRP, Dako) and diaminobenzidine for 10 minutes (DakoCytomation). Counterstain was carried out with hematoxylin for 10 minutes.

### Immunoprecipitation and mass spectrometry analyses

Cell lysates prepared from MCF7 breast cancer cells that endogenously express MUC1-ARF were subjected to immunoprecipitation using ProteinA/ProteinG agarose beads (Santa Cruz Biotechnology, catalog number sc-2002) to which the monoclonal antibody MPR2G10 had been prebound. Following extensive washing of the beads, bound proteins underwent trypsin digestion. Liquid chromatography–tandem mass spectrometry (LC–MS/MS) was performed using a 15 cm reversed-phase fused-silica capillary column (inner diameter, 75 um) made in-house and packed with 3 um ReproSil-Pur C18AQ media (Dr. Maisch HPLC GmbH).The LC system, an UltiMate 3000 (Dionex) was used in conjunction with an LTQ Orbitrap XL (Thermo Fisher Scientific) operated in the positive ion mode and equipped with a nanoelectrospray ion source. Peptides were separated with a four hour gradient from 5 to 65% acetonitrile (buffer A, 5% acetonitrile, 0.1% formic acid and 0.005% TFA; buffer B, 90% acetonitrile, 0.2% formic acid and 0.005%TFA). The voltage applied to the union to produce an electrospray was 1.2 kV. The mass spectrometer was operated in the data-dependent mode. Survey mass spectrometry scans were acquired in the Orbitrap with the resolution set to a value of 60,000. The seven most intense ions per scan were fragmented and analyzed in the linear ion trap. Raw data files were searched with MASCOT (Matrix Science) against a Swissprot database. Search parameters included a fixed modification of 57.02146 Da (carboxyamidomethylation) on Cys, and variable modifications 15.99491 Da (oxidation) on Met, and 0.984016 Da (deamidation) on Asn and Gln. The search parameters also included: maximum 2 missed cleavages, initial precursor ion mass tolerance 10 ppm, fragment ion mass tolerance 0.6 Da. Samples were further analyzed in Scaffold (Proteome software).

### Flow cytometry

After trypsinization, cells were washed, and mouse 2G10 antibody, with or without competitor peptide was added for 1 hour at 4°C. Following washing with flow cytometry (FACS) buffer, fluorescein labeled goat anti-mouse IgG antibody was added for 45 minutes at 4°C. Detection of bound IgG was by flow cytometry on a FACSCalibur^™^ (Becton Dickinson).

### Sandwich ELISA for determining MUC1-ARF/MUC1-TM levels

Elisa Immunoassay plates (CoStar) were coated with either MPR-2G10 mAb or with H23 mAb for detection of MUC1-ARF or MUC1-TM respectively, followed by blocking. Cell lysates at 1mg/ml protein concentration were then applied to the wells followed by adding anti-MUC1-ARF monoclonal antibody MPR-4B3, or anti-MUC1-TM monoclonal antibody H23, both conjugated to biotin. Detection of MUC1-ARF binding was performed by horseradish peroxidase (HRP)-conjugated to streptavidin. The results were calculated as an average of 3 experiments, performed in triplicates.

### In vitro transcription and translation

In vitro transcription and translation reactions were performed as previously described [18] and in vitro translation products were analyzed on standard SDS-polyacrylamide gels.

## Results

### Translation of MUC1-TM mRNA results in synthesis of both MUC1-TM ‘parent’ protein and an alternately-read MUC1-ARF protein

**Fig 1:**
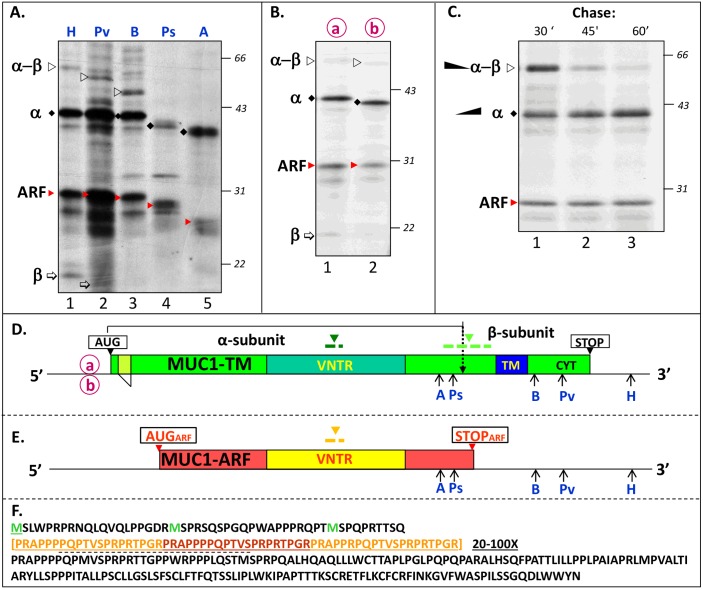
In vitro translation of MUC1 mRNA yields both MUC1-TM protein and MUC1-ARF protein. (A) RNAs transcribed from MUC1 cDNA [comprising a single 60 nucleotide (20 amino acid) repeat unit] cut with HindIII, PvuII, BalI, PstI or AccI (H, Pv, B, Ps and A, lanes 1–5 respectively, see Fig 1D and E) were translated in vitro with [^35^S]-methionine and [^35^S] cysteine and the products resolved by SDS-PAGE. Molecular masses of protein markers run in a parallel gel are indicated to the right of the autoradiogram, and are also shown in (B) and (C). (B) MUC1 mRNAs circled in red lettering a and b, that differ from each other by 27 nucleotides downstream from the MUC1-TM initiation codon (Fig 1D, a and b) were translated in vitro with [^35^S]-methionine and [^35^S]-cysteine and the products resolved by SDS-PAGE. (C) MUC1 mRNA containing a single 20 amino acid repeat was translated for 30 minutes in an in vitro reticulocyte protein translation system with [^35^S]-methionine and [^35^S]-cysteine and labelled proteins chased with an excess of unlabeled methionine and cysteine. Samples were removed (times indicated) and resolved by SDS-PAGE. (D) Locations of the initiating (AUG) and terminating (STOP) codons in the MUC1 mRNA driving translation of MUC1-TM are as indicated. The TransMembrane (TM) and cytoplasmic (CYT) domains, Variable Number of Tandem Repeats (VNTR) of MUC1-TM are as indicated. (E) MUC1-ARF, lacking these domains, is shown by VNTR (yellow color) flanked by 5' and 3' domains (red color). The initiation and stop codons (AUGARF and STOPARF) are shown (boxed). Regions bound by anti-MUC1-TM tandem repeats antibodies, anti-MUC1-ARF tandem repeats antibodies and anti-MUC1-SEA module antibodies (in MUC1-TM) are indicated by downward facing dark green, orange and light green arrowheads, respectively. (F) Amino acid sequence of MUC1-ARF. The MUC1-ARF twenty amino-acid-long repeat sequence is shown as three repeats (light and dark brown fonts) whereas in the actual MUC1-ARF protein the number of repeats may vary between fifteen and one hundred twenty five. The peptide sequence used for generating anti-MUC1-ARF monoclonal antibodies is underlined (dashed).

Translation of mRNA transcribed from MUC1 cDNA comprising one 60 nucleotide repeat unit revealed the expected MUC1-TM protein products, namely uncleaved MUC1 alpha-beta indicated by unfilled arrow-head, the MUC1-TM alpha-subunit indicated by [alpha, black diamond], and the C-terminal MUC1-TM beta-subunit indicated by [beta, unfilled arrow] (Fig 1A, lanes 1–3, and Fig 1B, lanes 1 and 2). The MUC1 mRNA used here for the in vitro translation assays contained only a single 60 nucleotide repeat unit, as compared to the 15–125 tandem repeat units present in naturally occurring MUC1 mRNA, thus explaining the relatively low molecular masses observed in the in-vitro translation assays both for the MUC1 alpha-beta parental protein and the MUC1-TM alpha-subunit. That the band labeled by [alpha, black diamond] is in fact the N-terminal MUC1-TM alpha-subunit has been previously unequivocally demonstrated [18]. Specifically, (i) it is *not* radioactively labeled when cysteine is used as the sole radioactive amino acid in the in-vitro translation reaction [see Fig 1 in [18]], in accord with the absence of cysteine residues in the MUC1-TM alpha-subunit and (ii) as expected for the MUC1-TM alpha-subunit formed by the ongoing cleavage within the MUC1 SEA module, in a pulse chase experiment the intensity of the [alpha, black diamond] band increases with the efflux of time [Fig 1C, and see Fig 1 in [18]]. The band migrating with a molecular mass of about 20kDa and here indicated by [beta, unfilled arrow] (Fig 1A, lanes 1 and 2, and Fig 1B, lanes 1 and 2) has been previously unambiguously identified as the MUC1-TM beta-subunit [18] and reconfirmed here, because (i) the 20kDa band is absent following translation of a MUC1 mRNA truncated at both the Pst1 and Acc1 sites that are located upstream to the N-terminus of the region coding for the MUC1-TM beta-subunit [compare Fig 1A, lanes 4 and 5 with Fig 1A lane 1, and see Fig 1 in [18]], and (ii) during the chase period of a pulse-chase experiment the levels of this protein also increase with time as does the MUC1-TM alpha-subunit [see Fig 1 in [18]]. Note that translation of MUC1 mRNA truncated at the PvuII site (see Fig 1D for the location of the PvuII site within the region coding for the cytoplasmic domain of the MUC1-TM beta-subunit) leads to a faster migrating band representing the curtailed MUC1-TM beta-subunit (Fig 1A, compare lane 2 with lane 1), whereas no alternative fragment was observed following translation of MUC1 mRNA truncated at the BalI site (Fig 1A, lane 3). This is because the fragment of the MUC1-TM beta-subunit derived from translation of MUC1 mRNA truncated at the BalI site is much shortened and therefore likely comigrates with the globin present in great amounts in the reticulocyte lysate. In order to allow for better resolution of the MUC1 alpha-beta parental protein, the MUC1-TM alpha-subunit and the MUC1-ARF protein (see below), electrophoresis in Fig 1C was performed for an extended period of time leading to elution from the gel of the small MUC1-TM beta-subunit.

Unexpectedly, following in-vitro translation of MUC1 mRNA an additional prominently labeled band designated MUC1-ARF (indicated by [red arrow-head], Fig 1A, lanes 1–3, Fig 1B and 1C) was observed. It migrated with a molecular mass in the region of 30kDa, and as described above (and see below for a more detailed analysis), it represents neither the MUC1-TM alpha-subunit nor the MUC1-TM beta-subunit. The fact that in-vitro translation of MUC1 mRNA truncated at sites *upstream* to the C-terminal end of the MUC1-TM alpha-subunit [for location of restriction sites see Fig 1D and 1E, AccI (A) and PstI (Ps),] leads to synthesis of truncated forms of the MUC1-ARF protein (Fig 1A, lanes 4 and 5, indicated by red arrow heads)) clearly indicates two points: (i) MUC1-ARF cannot possibly represent the MUC1-TM beta-subunit, because the MUC1 mRNAs truncated either at the AccI or at the Pst1 sites do not comprise information coding for MUC1-TM beta-subunit, and (ii) MUC1-ARF must be coded for by the MUC1 mRNA. Comparison of the two protein products, MUC1-ARF and the MUC1-TM alpha-subunit (compare ARF protein [red arrow-head] with that of the MUC1-TM alpha-subunit designated by [black diamond], Fig 1A, 1B and 1C), shows that in vitro synthesis results in MUC1-ARF protein levels that are only slightly less than those of the MUC1-TM alpha-subunit, indicating that translation of MUC1 mRNA yields significant amounts of MUC1-ARF protein. Translation of mRNAs generated from MUC1 cDNAs digested by restriction enzymes cleaving at sites upstream to the C-terminal end of the MUC1-TM alpha-subunit [Fig 1D and 1E, AccI (A) and PstI (Ps)] lead as expected to synthesis of C-terminally truncated MUC1-TM alpha-subunits (Fig 1A, bands designated by [black diamond]; compare lanes 4 and 5 with lanes 1, 2 and 3). Translation of the same truncated MUC1 mRNAs yielded MUC1-ARF proteins that are similarly diminished in size (Fig 1A, bands designated by [red arrow-head]; compare lanes 4 and 5 with lanes 1, 2 and 3) confirming that MUC1-ARF proteins do in fact derive from translation of MUC1 mRNA, as described above. As expected, translation of mRNAs generated from MUC1 cDNAs digested by PvuII (Pv) and BalI (B) that cut sites located upstream to the stop codon of *full-length* MUC1 yet downstream to the C-terminal end of the MUC1-TM alpha-subunit (see Fig 1D and 1E, for restriction sites), lead to the generation of truncated uncleaved MUC1 alpha-beta (Fig 1A, lanes 2 and 3, indicated by [unfilled arrowhead]), and intact MUC1-TM alpha-subunit and MUC1-ARF protein (Fig 1A, lanes 2 and 3, indicated by [black diamond] and [red arrow-head], respectively).

These results support the following conclusions: [1] The comparable decrease in molecular mass of the MUC1-TM alpha-subunit protein and of the MUC1-ARF protein arising from 3' truncation in the MUC1-TM mRNAs (Fig 1A, bands designated by [black diamond] and [red arrow-head], compare lanes 4 and 5 with lanes 1, 2 and 3) indicates that both proteins are translated from the same mRNA and terminate in proximity to each other. [2] Because the MUC1-TM mRNA in these experiments contained only a single tandem repeat sequence, the MUC1-ARF protein cannot represent polymorphic MUC-TM proteins varying in the numbers of tandem repeats, but in fact represents a distinct MUC1 protein entity. [3] The size difference between the MUC1-TM alpha-subunit proteins and the MUC1-ARF proteins (Fig 1A, 1B and 1C bands designated by [black diamond], and [red arrow-head] respectively) suggest that the two proteins are different.

Translation of two naturally occurring isoforms of MUC1-TM mRNA (‘a’ and ‘b’ in Fig 1, panel D), yields uncleaved full-length MUC1-TM protein and N-cleavage proteins that differ by about 1000 Daltons [alpha-beta (unfilled arrow-head) and alpha (black diamond), respectively Fig 1B, lanes 1 and 2). This is because variant ‘a’ differs from variant 'b' in that it comprises an additional 27 nucleotides coding for nine amino acids downstream to the AUG initiation codon of MUC1-TM. In contrast, because the two differently sized MUC1-TM mRNAs, 'a' and 'b', yield identically sized MUC1-ARF proteins [Fig 1B, lanes 1 and 2, compare MUC1-ARF proteins indicated by (red arrow-head)], we conclude that the initiation codon directing MUC1-ARF synthesis is located downstream to that initiating the MUC1-TM protein. Finally, because of the autoproteolytic cleavage of the precursor MUC1-TM protein within its SEA module into cleaved alpha- and beta-subunits, a pulse-chase experiment demonstrated a time-dependent decrease in levels of the full-length MUC1-TM protein (alpha-beta and designated by open arrow-head, Fig 1C, lanes 1–3), accompanied by an associated increase in MUC1-TM alpha-subunit (designated by alpha, black diamond, Fig 1C, lanes 1–3). In contrast, MUC1-ARF levels remained constant throughout the chase (indicated by ARF, red arrow-head, Fig 1C, lanes 1–3), demonstrating that MUC1-ARF clearly does not contain a cleavable SEA module and must represent a protein distinct from MUC1-TM.

**Fig 2:**
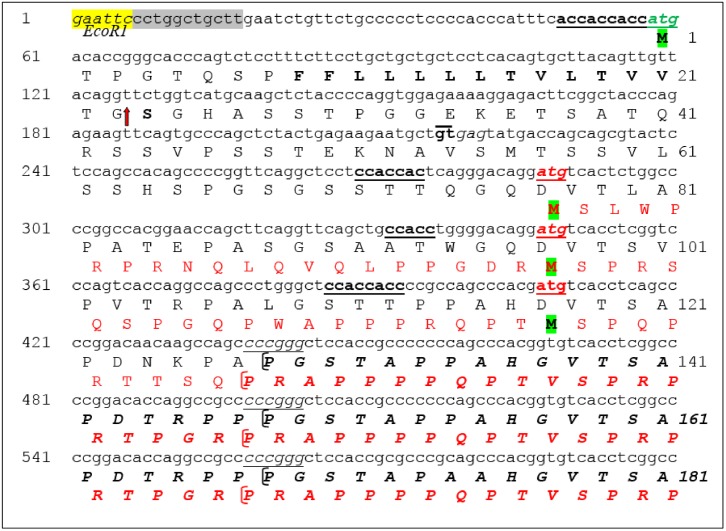
Nucleotide and amino acid sequence of MUC1-TM and MUC1-ARF. MUC1-TM initiation codon is shown in black font and green highlight. Downstream to this initiation codon, three potential MUC1-ARF initiation codons in a +1 frame are shown in red font and green highlight. Amino acid sequences are in black and red beneath the nucleotide sequence, represent MUC1-TM protein and MUC1-ARF protein, respectively. The red arrow indicates the signal peptide cleavage site of the MUC1-TM protein. Kozak sequences upstream to the initiation codons of both proteins are in bold fonts and underlined. The sequences shown for both MUC1-TM and MUC1-ARF extend from their respective initiation codons to their tandem repeat domains; three such repeats are shown indicated by square brackets. The EcoR1 site and grey highlighted regions are not part of the actual MUC1 cDNA sequence.

Analysis of the MUC1 mRNA reveals an AUG codon located 265 nucleotides downstream to the MUC1-TM initiation codon that (i) initiates a long +1 frameshifted open reading frame, (ii) contains an upstream Kozak consensus sequences (ccaccacc/t), and (iii) has the potential to initiate translation of a frameshifted alternate reading frame (ARF) protein (Fig 2). As shown above, in vitro translation of MUC1 mRNA yields proteins consistent with translation of the MUC1-TM mRNA in an alternative reading frame directed by an initiation codon downstream to that initiating MUC1-TM synthesis. Like MUC1-TM, MUC1-ARF harbors a tandem repeat (VNTR) domain, but because of the +1 frameshift, the amino acid sequence of both the MUC1-ARF tandem repeat as well as the N- and C- sequences flanking it differ entirely from those of the MUC1-TM protein (Fig 1F). Because MUC1 mRNA comprising only *one* tandem repeat was used for the in vitro translation assays described above, the MUC1-ARF protein product (with one 20 amino acid repeat sequence) is predicted to contain 245 amino acids and the molecular mass for MUC1-ARF observed here (in the region of 30kDa) corresponds well with that expected for the in-vitro translated MUC1-ARF protein.

Remarkably, the alternate reading frame codes for 49 amino acids upstream to the VNTR and 175 amino acids downstream to the VNTR. Assuming a minimum of about 15 tandem repeats for a naturally occurring MUC1 mRNA, each comprising twenty amino acids [19], the VNTR of MUC1-ARF alone contains at least 300 amino acids, and the complete MUC1-ARF protein would then comprise at least 524 amino acids.

### MUC1-ARF protein in MUC1 transfected cells

**Fig 3:**
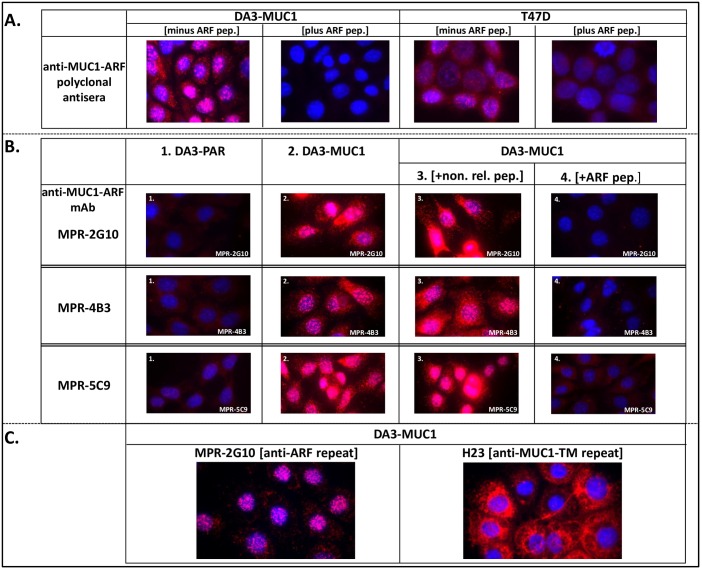
Detection of nuclear MUC1-ARF protein with polyclonal anti-MUC1-ARF antibodies and with three distinct anti-MUC1-ARF monoclonal antibodies, MPR2G10, MPR4B3 and MPR5C9. (A) Polyclonal anti-MUC1-ARF antibodies and (B) three independently isolated anti-MUC1-ARF monoclonal antibodies, MPR2G10, MPR4B3 and MPR5C9, were reacted in the presence of competing ARF peptide (B, panels 4), in its absence (B, panels 2), or with a non-relevant peptide (B, panels 3) with mouse DA3 cells stably transfected with and expressing human MUC1 DNA (DA3-MUC1) and with T47D human breast cancer cells that endogenously express MUC1. Parental DA3 cells (DA3-PAR) which do not express human MUC1 are shown in B, panels 1. Immunofluorescence of secondary antibody (red), DAPI staining of nuclei (blue) are shown in the merged images. (C) Mouse DA3 cells expressing human MUC1 reacted with anti-MUC1-ARF repeat monoclonal antibody MPR2G10 (left panel), compared with anti-MUC1-TM tandem repeat monoclonal antibody H23 (right panel), both followed by red-labelled secondary antibody.

**Fig 4:**
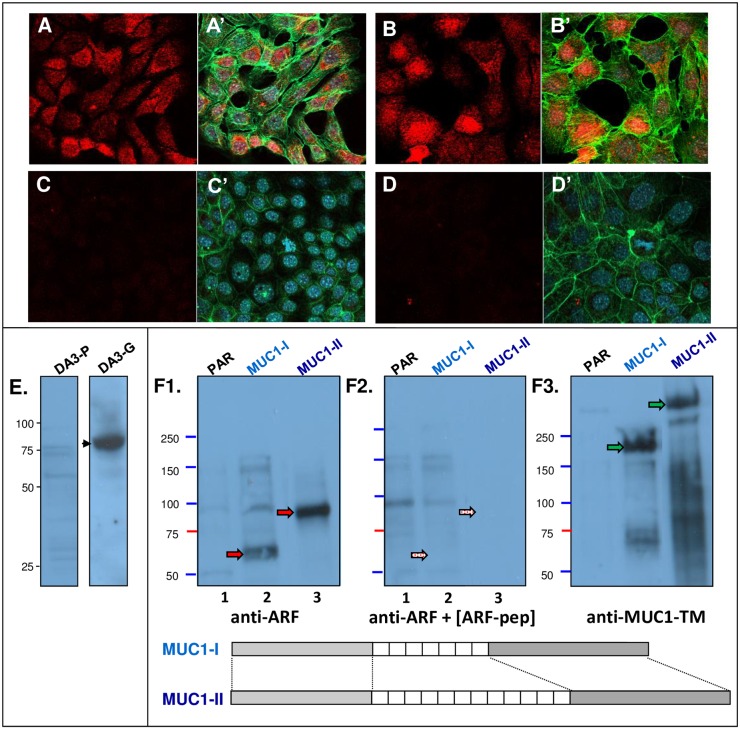
Mouse cell lines transfected with human MUC1 DNA express MUC1-ARF. (A, and C) DA3 cells transfected with MUC1 cDNA; (B) DA3 cells transfected with human MUC1 genomic DNA, and (D) non-transfected mouse parental DA3 cells were immunostained with anti MUC1-ARF monoclonal antibody MPR2G10 followed by red-labeled secondary antibody. DAPI (blue) and green-labeled phalloidin are shown as merged images. In (C), anti-MUC1-ARF monoclonal antibody was added together with competing ARF peptide. (E) Immunoblots with anti-MUC1-ARF antibodies of cell lysates prepared from either untransfected DA3 mouse cells (DA3-P) or from cells transfected with MUC1 genomic DNA (DA3-G). MUC1-ARF is indicated by the arrowhead. (F) Mouse 3T3 cells transfected with human MUC1 DNA containing differently sized VNTRs (diagrams designated MUC1-I, MUC1-II) express differently sized MUC1-ARF proteins: Cell lysates from either parental 3T3 cells or 3T3 transfectants stably expressing human MUC1 DNA (lanes 1, 2 and 3, designated PAR, MUC1-I and MUC1-II, respectively) were resolved by SDS-PAGE and immunoblots probed with anti-MUC1-ARF alone (F1) or together with competing ARF peptide (F2). Following probing, the immunoblot was stripped, reprobed with an antibody recognizing an epitope within the MUC1-TM tandem repeat sequence, and redeveloped with ECL (F3). Filled red arrows indicate MUC1-ARF proteins and filled green arrows indicate MUC1-TM proteins (F1 and F3, respectively). Stippled red arrows designate the positions of the MUC1-ARF proteins that have been specifically competed out. Protein loading with anti-actin antibodies confirmed that equal amounts of protein were present in each lane. MUC1-I and MUC1-II cDNAs used for transfections contains approximately 17 and 28 repeats respectively. The two cartoons at the bottom of the Figure designating the MUC1-I and MUC1-II cDNAs are intended for illustrative purposes only. They demonstrate the fact that the two cDNAs differ one from the other in the number of repeats each contains.

We investigated MUC1-ARF expression with anti-MUC1-ARF monoclonal antibodies (mAbs) generated by immunizing mice with a peptide representing 1.3 repeat units of the MUC1-ARF tandem repeat (PQPTVSPRPRTPGRPRAPPP-PQPTVS- one 20 amino acid repeat is underlined and six amino acids of the following repeat is double-underlined (Fig 1, panel F). Screening against the MUC1-ARF repeat peptide yielded three independent hybridomas secreting monoclonal antibodies MPR2G10, MPR4B3 and MPR5C9. Following reaction of each of the three antibodies with mouse mammary tumor cells stably transfected with and expressing human MUC1 DNA (DA3-MUC1) a strong nuclear signal was seen, as well as a lower level signal in the cell cytoplasm (Fig 3B Panels 2, DA3-MUC1). Parental mouse DA3 cells not expressing the human MUC1 protein, were uniformly non-reactive with the anti-MUC1-ARF monoclonal antibodies (Fig 3B Panels 1, DA3-PAR). Furthermore, addition of MUC1-ARF peptide abrogated the immunoreactivity of the antibodies, whereas addition of a non-relevant peptide had no effect (Fig 3B, DA3-MUC1, compare panels 4 and 3). Similar results were obtained with anti-MUC1-ARF polyclonal antisera, reacted with both DA3-MUC1 cells as well as with T47D breast cancer cells (Fig 3A). As previously reported [16, 20–22], cells transfected with human MUC1 DNA express high levels of the human MUC-TM protein as assessed with anti-MUC1-TM tandem repeat mAbs that localized exclusively to the cell membrane and cytoplasm (Fig 3C, right-hand panel). Reacting the same cells in parallel with anti-ARF-repeat mAb MPR2G10 demonstrated predominantly nuclear localization (Fig 3C, left-hand panel), consistent with the pattern shown above. A sandwich ELISA assay consisting of capture mAb MPR2G10 and detecting biotinylated mAb MPR4B3 could readily detect the MUC1-ARF protein (see below section **"Endogenous MUC1-ARF protein is detected in human cancer cell lines**"), and although the anti-MUC1-ARF mAbs recognize discrete epitopes in the MUC1-ARF repeat sequence, they all bind to the MUC1-ARF protein. Immunoreactivity of anti-MUC1-ARF mAb MPR2G10 with DA3 cells transfected with both human MUC1 cDNA and human genomic MUC1 DNA was the most robust (Fig 4, panels A and B). It was therefore chosen for subsequent studies. Probing immunoblots of lysates from cells transfected with human MUC1 DNA demonstrated MUC1-ARF that migrated with a molecular mass of about 80kDa (Fig 4E, lane DA3-G). MUC1-ARF protein was seen neither in non-transfected cells (Fig 4E, lane DA3-P), nor following addition of competing ARF peptide.

Two distinct murine 3T3 transfectants, each receiving human MUC1 cDNA comprising a different number of tandem repeats provided further confirmation of MUC1-ARF expression in MUC1-transfected cells. Variation in the number of tandem repeats provides an inherent size-signature ‘barcode’ to MUC1 proteins translated by each of the two transfectants. Accordingly, when western blots were probed with anti-[MUC1-TM tandem repeat] antibodies, each of the two stable 3T3 transfectants showed differently sized high molecular mass MUC1-TM proteins (Fig 4F3, filled green arrows), reflecting the size of the tandem repeat domain contained within each of the MUC1 cDNAs used for transfection. MUC1-TM glycoprotein is heavily post-translationally modified by both N- and O-linked glycosylations [14, 23, 24]. Probing similar blots with anti-MUC1-ARF revealed that the two transfectants produced distinctly sized MUC1-ARF proteins (Fig 4F1, filled red arrowheads). Cells transfected with MUC1-I or MUC1-II cDNA expressed MUC1-ARF proteins of approximately 60kDa and 90kDa respectively, corresponding to the tandem repeat polymorphisms of the transfected MUC1 DNAs. The calculated mass of the MUC1-ARF protein with a single repeat unit is 27,253 Daltons. Each additional MUC1-ARF repeat unit adds on an additional calculated mass of 2,161.5 Daltons. We therefore estimate that the MUC1-I and MUC1-II cDNAs comprise about 17 and 28 MUC1-ARF repeat units, respectively. Addition of competing MUC1-ARF peptide ablated immunoreactivity, confirming the specificity of the anti-MUC1-ARF antibodies (Fig 4F2, lanes 2 and 3) as does the absence of reactivity in lysates from non-transfected cells (Fig 4F1, lane 1, designated PAR).

Since the molecular mass of MUC1-ARF protein correlates with the number of tandem repeat sequences contained within the *MUC1* gene, which varies between different individuals and cell lines, the size of MUC1-ARF proteins will correspondingly vary. This means that differently sized MUC1-ARF proteins will be detected in different cell lines.

To see whether MUC1-ARF has the potential to be post-translationally modified, the MUC1-ARF amino acid sequence was interrogated using the MotifScan analysis (http://scansit.mit.edu). In order to improve our level of confidence in the analysis, the scan was performed *only at the* ***highest*** *level* of stringency and we purposely did not opt for the medium and low stringency levels of scanning. This MotifScan analysis predicted, with very high levels of confidence, multiple sites for post-translational modifications within the MUC1-ARF protein. Sites for phosphorylation on serine or/and threonine (***S/T***) residues mediated by calmodulin dependent kinase 2 (CAMGK2G) were predicted in the following sequences: RQP***T***MSP located just upstream to the tandem repeats, RPR***T***PGR and PTV***S***PRP both sites located within the tandem repeats, and PMV***S***PRP and YLL***S***PPP, sites located C-terminal to the tandem repeats (***T*** or ***S*** phosphorylation sites are in bold and italics).

The molecular masses of the two MUC1-ARF proteins (Fig 4F1, lanes 2 and 3) are considerably less than those of the corresponding MUC1-TM proteins (Fig 4F3, filled green arrows), suggesting that although the MUC1-ARF proteins are likely post-translationally modified, as described above, these are not as extensive as those of MUC1-TM.

### Endogenous MUC1-ARF protein is detected in human cancer cell lines

**Fig 5:**
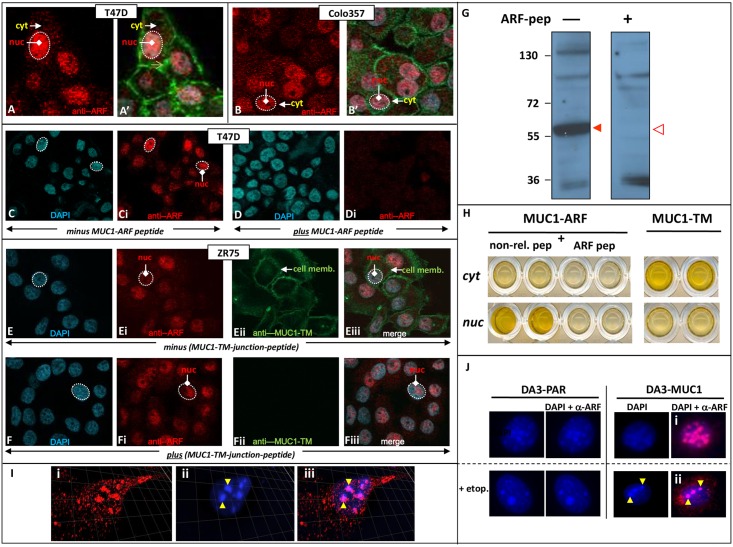
Human cancer cells express MUC1-ARF in both the nucleus and cytoplasm. T47D breast cancer cells (Panels A and C) and COLO357 pancreatic cancer cells (Panels B) were immunostained with anti-MUC1-ARF mAb MPR2G10 followed by red-labeled secondary antibody. DAPI (blue, demonstrating nuclei: white stippled ovals) and green-labeled phalloidin (labeling actin filaments) are shown in the merged images (Panels A' and B'). Immunostaining of T47D cells with anti-MUC1-ARF mAb MPR2G10 is abrogated when done in the presence of competing MUC1-ARF peptide (compare Panels C with Panels D). Simultaneous immunostaining of ZR75 breast cancer cells with anti-MUC1-TM antibodies (DMB5F3 mAbs directly green labeled) and anti-MUC1-ARF antibodies (MPR2G10, red labeled) in the absence of MUC1-TM-junction peptide is shown in Panels E, while the effect of adding MUC1-TM-junction peptide to an identical immunostaining of ZR75 breast cancer cells is shown in Panels F. (Panel G) Lysates of human COLO357 cancer cells were resolved on SDS-PAGE, western blotted, and probed with anti-MUC1-ARF MPR2G10. MUC1-ARF protein is indicated by the filled red arrow head (left panel). Immunoreactivity is abrogated by addition of competing free MUC1-ARF peptide (right panel). (Panel H, left side, labeled MUC1-ARF) Equivalent amounts of protein from either cytoplasmic or nuclear (cyt or nuc) T47D cell extracts were analyzed by a sandwich ELISA that detects MUC1-ARF. Competing MUC1-ARF peptide (ARF pep) added to the detecting biotinylated anti-MUC1-ARF MPR4B3, abolished signal in both cytoplasmic and nuclear samples, whereas non-relevant peptide (non-rel. pep) had no effect. (Panel H, right side, labeled MUC1-TM)- analysis of nuclear and cytoplasmic T47D cell extracts with a sandwich ELISA detecting MUC1-TM protein. (Panel I) Mouse DA3 mammary tumor cells expressing human MUC1 cDNA were immunostained with anti MUC1-ARF antibody MPR2G10 and red-labeled secondary antibody followed by DAPI staining. High magnification images of orthogonal projections of confocal laser microscopy are shown for anti-MUC1-ARF (Panel I-i), DAPI (Panel I-ii) and merged images (Panel I-iii). (Panel J) Untransfected mouse DA3 mammary tumor cells (DA3-PAR, left panels) or transfected with human MUC1 cDNA (DA3-MUC1, J, right panels) were stained with DAPI and immunostained with anti-MUC1-ARF monoclonal followed by red-labeled secondary antibody. DAPI staining alone (blue) and the merged images of DAPI plus red anti-ARF immunostaining (DAPI + anti-ARF) are shown. A parallel set of cells (Panel J, lower panels, plus etop.) were treated with Etoposide, a DNA topoisomerase II inhibitor.

We extended our analyses to human cancer cells which natively express high levels of the MUC1-TM protein. Immunofluorescent analyses done with anti-MUC1-ARF mAb 2G10 showed expression of endogenous MUC1-ARF protein that localized primarily to the cell nucleus. This was seen in T47D breast cancer cells (Fig 5, panels A and C), COLO357 pancreatic cancer cells (Fig 5, panels B) and ZR75 breast cancer cells (Fig 5, panels E and F). The prominent nuclear staining seen in T47D breast cancer cells with the anti-MUC1ARF mAb 2G10 was abrogated by addition of competing MUC1-ARF peptide (compare Fig 5, panels C and D), showing antibody specificity. Simultaneous costaining of breast cancer ZR75 cells (Fig 5, panels E) with anti-MUC1-TM mAbs [DMB5F3 [17], directly labeled with a green fluorescent dye] together with anti-MUC1-ARF mAb 2G10 (labeled red), clearly showed that the two MUC1 proteins did not colocalize: MUC1-TM localized at the cell membrane as expected, whereas MUC1-ARF protein was predominantly nuclear (Fig 5, see panels Ei, Eii and Eiii). Furthermore addition of competing MUC1-TM-junction protein abrogated the cell membrane signal of MUC1-TM, and was of no effect on the nuclear MUC1-ARF (Fig 5, compare panels E and panels F). Probing blots with anti-MUC1-ARF antibody 2G10 further demonstrated that endogenous MUC1-ARF protein migrated with a molecular mass of slightly more than 55kDa (Fig 5G, left lane), and that its reactivity was abolished by MUC1-ARF peptide (Fig 5G, open red arrow, right lane). The molecular mass of the protein seen in Colo357 cells corresponds to a MUC1-ARF protein containing about 14 MUC1-ARF repeat units.

**Fig 6:**
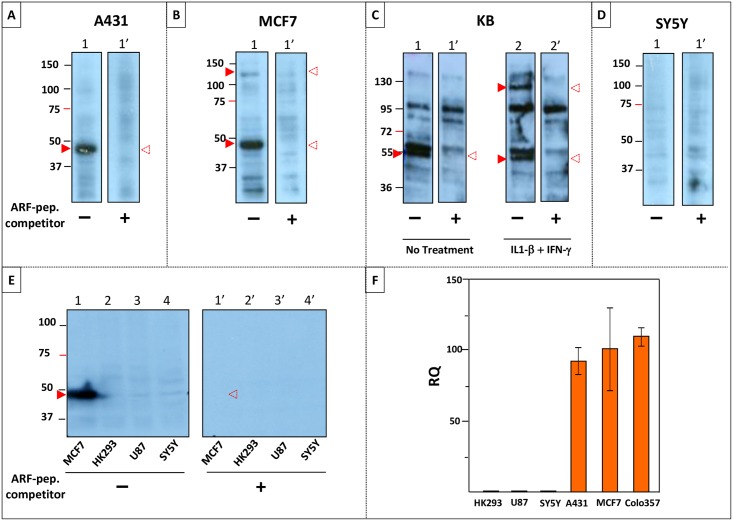
MUC1-ARF protein is observed only in those human cell lines that express the *MUC1* gene. Lysates prepared from human cell lines A431, MCF7, KB, SY5Y, HK293 and U87 (Panels 'A' to 'E') were resolved on SDS-PAGE, western blotted, and probed with anti-MUC1-ARF MPR2G10. MUC1-ARF protein is indicated by the filled red arrowheads. Addition of competing free MUC1-ARF peptide (indicated beneath the lanes by a 'plus' sign) competed out reactivity of the MUC1-ARF protein (white arrowheads with dotted red outline designate the positions of MUC1-ARF protein that has been competed out by added ARF peptide). (Panel F): Expression levels of the *MUC1* gene in the various cell lines were assessed in triplicate by quantitative PCR (qPCR). Expression in MCF7 cells was set to be 100. (Immunoblot analysis of Colo357 for MUC1-ARF expression appears in Fig 5G).

Human cancer cell lines A431, MCF7 and KB known to express MUC1-TM protein were assessed for MUC1-ARF expression by western blot analyses. Results indicate that cells which express MUC1 mRNA also expressed MUC1-ARF proteins with molecular masses ranging from about 48kDa to 55kDa (Fig 6, panels A, B and C, MUC1-ARF protein indicated by lower red arrowhead). These correspond to MUC1-ARF proteins containing about 11 to 17 MUC1-ARF tandem repeat units expressed by the relatively smaller MUC1 allele present in the these cell lines. A larger MUC1-ARF protein, expressed from the larger MUC1 allele detected in MCF7 cells migrated with a molecular mass of about 130kDa, corresponding to a MUC1-ARF protein containing about 47 ARF tandem repeat units (Fig 6B, upper red arrowhead). Its intensity was significantly less as compared to that of the small allele gene product (Fig 6B, lane 1, compare upper and lower red arrowheads), consistent with the previously reported reduced expression of the larger MUC1 alleles gene products [25]. In fact in the absence of added cytokines, only the smaller MUC1-ARF allele could be detected in KB cells (Fig 6C, lane 1 red arrowhead). However, addition of IL1-beta and interferon-gamma to KB cells, a combination known to increase MUC1 gene expression [26], led to the appearance of the larger MUC1-ARF protein (Fig 6C lane 2, indicated by upper red arrowhead with a mass of about 120kDa) corresponding to the larger MUC1 allele present in these cells. The size difference of the two MUC1-ARF proteins observed in KB cells tallies remarkably well with that published for the two MUC1 alleles of KB cells as determined by Northern blotting analyses [26].

The reactivity of the MUC1-ARF protein was in all cases abolished by the addition of competing MUC1-ARF peptide [Fig 6A, 6B and 6C, compare lanes 1 (minus competing MUC1-ARF peptide) with lanes 1' (plus competing MUC1-ARF peptide); Fig 6C, compare lanes 2 and 2'; red arrowheads indicate the MUC1-ARF proteins and white arrowheads with dotted red outline designate the position of MUC1-ARF protein competed out by added ARF peptide]. No MUC1-ARF protein was detected in the neuroblastoma cell line SY5Y consistent with the undetectable expression of the *MUC1* gene in these cells (Fig 6D and 6F). An additional two cell lines, HK293 and U87, which do not express the *MUC1* gene, also did not express the MUC1-ARF protein (Fig 6E).

Quantitative PCR analyses (qPCR) to assess expression of MUC1 mRNA validated that only those cells (A431, MCF7 and Colo357) that express MUC1 mRNA also displayed MUC1-ARF protein, whereas those cells that had low to undetectable levels of MUC1 expression (HK293, U87 and SY5Y) were likewise negative for MUC1-ARF protein (Fig 6A–6F). Taken together, these analyses confirm endogenous MUC1-ARF protein in human cancer cell lines known to express the *MUC1* gene, and conversely the absence of MUC1-ARF protein in cells that do not express the *MUC1* gene.

Quantitation of MUC1-ARF protein in nuclear and cytoplasmic compartments demonstrated MUC1-ARF protein in nuclear extracts prepared from human T47D breast cancer cells while cytoplasmic extracts displayed much lower levels (Fig 5H, left panel). Analysis of MUC1-TM revealed an inverse picture: while high levels were observed in cytoplasmic extracts, nuclear MUC1-TM was almost undetectable (Fig 5H, right panel). Within the nucleus itself MUC1-ARF staining revealed a non-homogeneous distribution, with intense MUC1-ARF immunostaining observed in discrete subnuclear aggregates (red staining, Fig 5A, 5B, 5C-i, 5E-i and 5F-i). Additional analyses by confocal laser microscopy and orthogonal projections (Fig 5I, 5i, 5ii and 5iii) showed intense staining with both DAPI and with anti-MUC1-ARF antibodies, suggesting that MUC1-ARF localizes to subnuclear regions comprising condensed chromatin. This colocalization was further confirmed by etoposide treatment of MUC1 cDNA-transfected cells which induced large DAPI-positive condensed chromatin aggregates to which MUC1-ARF clearly colocalized (Fig 5J, compare panels i and ii). In contrast, immunostaining of non-transfected cells was negative (Fig 5J, DA3-PAR).

### Expression of MUC1-ARF in normal human tissues correlates with MUC1-TM expression

**Fig 7:**
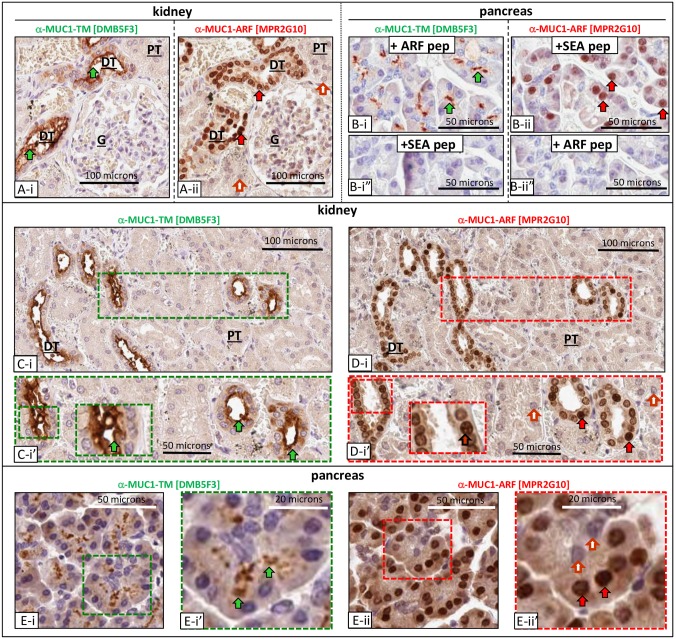
MUC1-TM and MUC1-ARF expression in normal human kidney and pancreas. Serial sections of paraffin-embedded human pancreatic and renal tissues were immunohistochemically stained with anti-MUC1-TM SEA module antibodies (anti-MUC1-TM [DMB5F3]) and anti-MUC1-ARF antibodies (anti-MUC1-ARF [MPR2G10]) as indicated. Normal kidney is shown in Panels A-i (MUC1-TM) and A-ii (MUC1-ARF); larger fields and higher magnifications are shown in Panels C-i, C-i’ (MUC1-TM), and D-i, D-i’ (MUC1-ARF)]. Glomerulus, proximal tubule, and distal tubule are designated by **G**, **PT** and **DT** respectively. Filled green arrows designate sites of MUC1-TM-SEA protein at the cell surface whereas filled red arrows designate MUC1-ARF protein; absence of anti-MUC1-ARF immunoreactivity is shown by filled red and white arrows. Normal pancreatic tissue reacted with anti-MUC1-TM in the presence of ARF peptide (plus ARF pep) or in the presence of MUC1-TM SEA peptide (plus SEA pep), are shown in Panels B-i and B-i", respectively. MUC1-TM protein on the cell surface of ductal epithelial cells is competed out by MUC1-TM SEA peptide (B-i") but not by MUC1-ARF peptide (B-i). Conversely MUC1-ARF protein in the nuclei of pancreatic epithelial cells is competed out by MUC1-ARF peptide (B-ii") but not by MUC1-SEA peptide (B-ii). Larger fields and higher magnifications of pancreatic tissue is shown in E-i, E-i’ (MUC1-TM), and E-ii, E-ii’ (MUC1-ARF).

MUC1-TM is detected on the apical surface of normal epithelial cells that form luminal structures (for example, [27]). To see whether MUC1-ARF protein is co-expressed with MUC1-TM in these cells, sequential serial sections of human tissues were reacted with monoclonal antibodies specific for either MUC1-TM or MUC1-ARF. To detect MUC1-TM we used the previously characterized mAb DMB5F3 [17] which binds specifically to the MUC1-TM SEA module with picomolar affinity. For MUC1-ARF detection, use was made of the mAb MPR2G10, here shown to bind the MUC1-ARF tandem repeat sequence (see Fig 1D and 1E for regions of MUC1-TM and MUC1-ARF recognized by the antibodies). In kidney tissue, MUC1-ARF expression was seen only in those cells that also expressed MUC1-TM [Fig 7, compare Panels A-i (MUC1-TM) with A-ii (MUC1-ARF)]. Larger fields and higher magnifications are shown in Fig 7; Panels C-i and C-i’ (MUC1-TM) and Panels D-i and D-i’ (MUC1-ARF)]. Similar results were seen with pancreatic sections (Fig 7, Panels B-i (MUC1-TM) with B-ii (MUC1-ARF)]. For larger fields and at higher magnifications see Fig 7, Panels E-i and E-i’ (MUC1-TM) and Panels E-ii and E-ii’ (MUC1-ARF). In contrast to MUC1-TM localization on the apical surfaces of normal luminal-forming epithelial cells, MUC1-ARF localized primarily to the nuclei of these same cells both in kidney and in pancreas. Addition of ARF peptide abrogated reactivity only of anti-MUC1-ARF antibody (Fig 7, compare Panel B-ii with B-ii"), whereas recombinant MUC1-SEA-module protein abrogated reactivity only of anti-MUC1-SEA antibody (Fig 7, compare Panels B-i with B-i"), confirming specificity of each antibody with its target protein.

Kidney tissue sections were particularly informative in that only luminal-forming cells of distal tubules (DT) stained positive for the MUC1-TM protein (Fig 7, Panels A-i, C-i and C-i') whereas cells of proximal tubules (PT), glomeruli (G), and Bowman’s capsule were uniformly negative. Correspondingly MUC1-ARF is expressed only in cells forming the lumen of distal tubules where it localized to cell nuclei, directly correlating with MUC1-TM expression (compare MUC1-ARF expression in Fig 7, Panels A-ii, D-i and D-i' with MUC1-TM expression in Fig 7, Panels A-i, C-i and C-i'). In cells of the exocrine pancreas most cells demonstrated nuclear MUC1-ARF expression, whereas a subset showed neither nuclear nor cytoplasmic MUC1-ARF (Fig 7, Panels B-ii, E-ii and E-ii').

### Expression of both MUC1-ARF and MUC1-TM expression is restricted to the exocrine pancreas and neither are expressed in the endocrine pancreas

**Fig 8:**
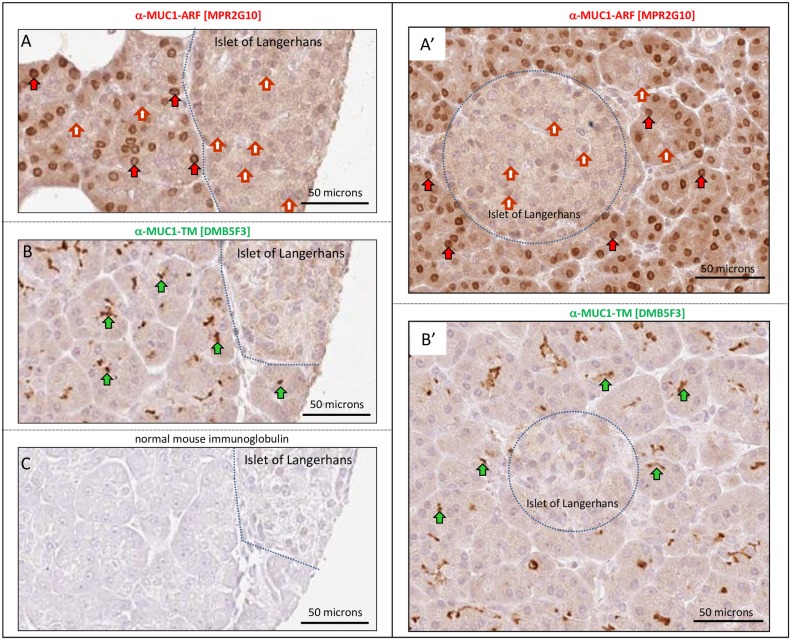
MUC1-TM and MUC1-ARF proteins are expressed solely in the exocrine pancreas and not in the endocrine pancreatic islets. Serial sections of paraffin-embedded human pancreatic tissues were immunohistochemically stained with anti-MUC1-TM SEA module antibodies (anti-MUC1-TM [DMB5F3]), anti-MUC1-ARF antibodies (anti-MUC1-ARF [MPR2G10]) and normal mouse immunoglobulin as indicated. Filled green arrows designate sites of MUC1-TM-SEA protein at the cell surface whereas filled red arrows designate MUC1-ARF protein; absence of anti-MUC1-ARF immunoreactivity is shown by filled red and white arrows. The pancreatic islets (Islets of Langerhans) are demarcated by the dotted blue lines.

Whereas MUC1-TM protein was readily observed at the apical surfaces of pancreatic ducts of the exocrine pancreas (Fig 8B and 8B'), no immunoreactivity for MUC1-TM was observed in the pancreatic islets forming the 'endocrine pancreas' (Fig 8B and 8B'- note the absence of MUC1-TM immunoreactivity in the Islets of Langerhans, demarcated by the blue dotted lines). This is in accord with the known expression of MUC1-TM solely within the exocrine pancreas, in cells lining the intercalated, intralobular and interlobular ducts. Cells of neuroendocrine origin forming the Islets of Langerhans are known not to express MUC1 [28–30]. MUC1-ARF protein was also exclusively expressed in cells of the exocrine pancreas where it localized primarily to cell nuclei (Fig 8A and 8A'). In contrast, and again in accord with MUC1-TM expression, all cells of the endocrine pancreas were uniformly negative for MUC1-ARF (Fig 8A and 8A'). These results demonstrate that (a) in the pancreas only those cells that express the MUC1-TM protein isoform also express MUC1-ARF, just as observed for kidney tissue, and (b) the nuclear staining for MUC1-ARF is specific because nuclei of cells comprising the Islets of Langerhans were uniformly negative for MUC1-ARF staining, whereas cells of the exocrine pancreas contained within the same section were MUC1-ARF positive.

Taken together, these studies show a tight correlation in normal tissues between MUC1-ARF expression and that of MUC1-TM. Certain cell types both in the kidney and pancreas that express MUC1-TM also express MUC1-ARF, whereas those kidney and pancreatic cells that do not express MUC1-TM, also do not express MUC1-ARF (Figs 7 and 8). Furthermore, normal tissues such as brain and liver that have no MUC1-TM expression are also devoid of MUC1-ARF protein.

### MUC1-ARF expression in normal and malignant pancreatic and breast tissues

**Fig 9:**
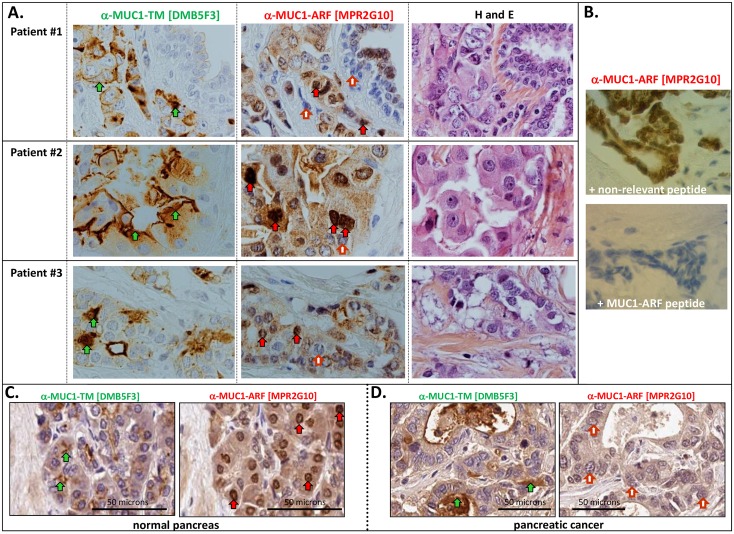
Immunohistochemical analyses of MUC1-TM and MUC1-ARF expression in breast and pancreatic cancer. (A) Serial sections of breast cancer tissues from three distinct individuals were immunohistochemically stained with anti-MUC1-TM antibodies (anti-MUC1-TM [DMB5F3]), anti-MUC1-ARF antibodies (anti-MUC1-ARF [MPR2G10]) and with hematoxylin/eosin (H and E). Green arrows indicate sites of MUC1-TM reactivity at the cell surface, and red arrows designate MUC1-ARF reactivity in the nuclei. Absence of anti-MUC1-ARF immunoreactivity is shown by filled red and white arrows. (B) Binding specificity of anti-MUC1-ARF [MPR2G10] antibody is demonstrated by addition of either MUC1-ARF peptide or a non-relevant peptide, as indicated. Only MUC1-ARF peptide abrogates immunoreactivity. Serial sections of normal pancreas (C) or pancreatic cancer tissue (D), were immunohistochemically stained with anti-MUC1-TM [DMB5F3] or anti-MUC1-ARF [MPR2G10]. Both MUC1-TM and MUC1-ARF are expressed in the normal pancreatic tissue (C). In contrast, cancer tissue expresses only MUC1-TM. MUC1-ARF was not detected (red and white arrows).

The patterns of MUC1-TM and MUC1-ARF expression in normal and malignant pancreatic and breast tissues displayed significant differences. High MUC1-TM expression was observed in pancreatic cancer tissues, accompanied by a loss of MUC1-ARF expression [compare expression of MUC1-TM and MUC1-ARF in normal pancreatic tissue (Fig 9C, left and right panels respectively), to that seen in pancreatic cancer tissue (Fig 9D, left and right panels respectively)]. In contrast to normal pancreatic tissues, both MUC1-TM and MUC1-ARF are expressed at very low levels in resting normal breast tissue. In breast cancer cells we found substantial MUC1-TM expression (Fig 9A, examples of expression in 3 breast cancer samples, left-hand panels), consistent with elevated MUC1-TM expression in breast tumors as described in the literature [14, 31]. Our analyses of a series of breast tumor tissues demonstrated that approximately 40% of the MUC1-TM positive sections (totaling 96 different samples) were MUC1-ARF positive. MUC1-ARF expressers (for examples see Fig 9A, middle panels, labeled anti-MUC1-ARF) could be roughly divided into three groups differing from each other in their patterns of MUC1-ARF expression: **[i]** high expression of both MUC1-TM and nuclear MUC1-ARF, **[ii]** high MUC1-TM expression and no MUC1-ARF expression, **[iii]** high MUC1-TM expression and focal MUC1-ARF expression in only a subset of cells. The significance of this MUC1-ARF expression in only some of the breast cancer samples is further considered in the Discussion section.

### Proinflammatory cytokines upregulate expression of MUC1-ARF protein

**Fig 10:**
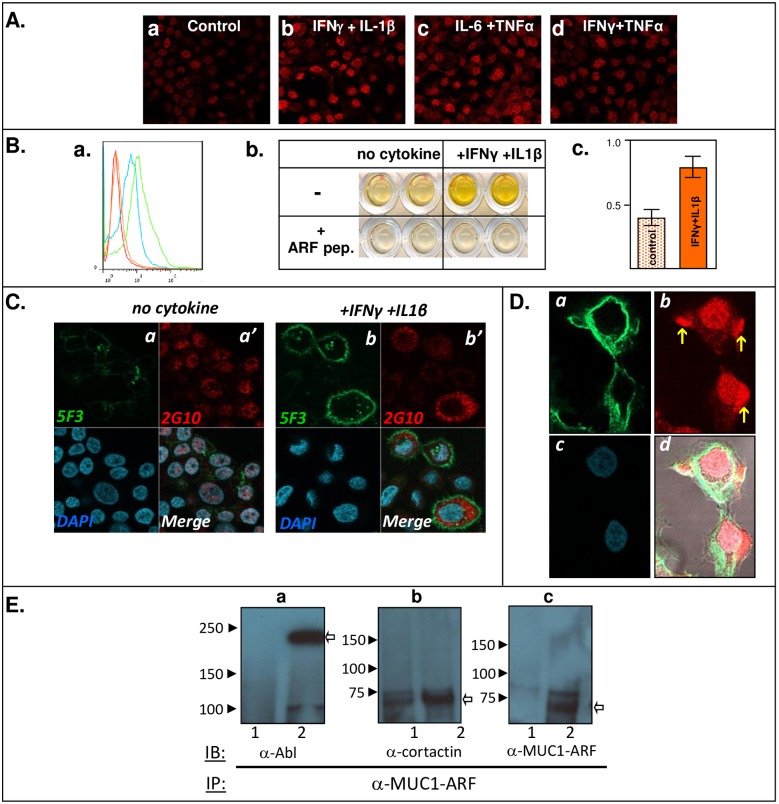
Cytokines upregulate MUC1-ARF expression and result in its relocalization. (A) COLO357 pancreatic cancer cells were either untreated (Control, Panel a), or incubated for 8 hours with interferon-gamma and interleukin1-beta (Panel b); interleukin 6 and TNFalpha (Panel c); interferon-gamma and TNFalpha (Panel d). This was followed by immunostaining with anti MUC1-ARF antibody MPR2G10 and red-labeled secondary detection. (B a.) COLO357 cells were either untreated or incubated for 8 hours with interferon-gamma and interleukin1-beta, (blue and green tracings, respectively), permeabilized, and analyzed by flow cytometry with anti-MUC1-ARF MPR-2G10, and detection with fluorescently labeled secondary anti-mouse antibody. Addition of secondary antibodies alone to untreated and cytokine-treated cells is shown by the orange and red tracings, respectively. (B b. c.) MUC1-ARF protein in untreated and cytokine-treated COLO357 cells was quantitatively assessed by sandwich ELISA (b), and shown in block graphs in (c). Specificity of antibody binding to MUC1-ARF protein was confirmed by addition of competing MUC1-ARF peptide (b. plus ARF peptide). (C) COLO357 cells were either untreated (Panels a, a', designated 'no cytokine') or incubated for 24 hours with interferon-gamma plus interleukin1-beta (Panels b, b', designated 'plus IFNgamma plus IL1beta'). Immunostaining with anti-MUC1-ARF MPR2G10 and detection by red-labeled secondary antibody (a' and b') was followed by green-labeled anti-MUC1-SEA module DMB5F3 (a and b). DAPI (blue) and merged images are presented in the lower left and right panels, respectively. (D) COLO357 cells were treated for forty-eight hours with IFNgamma and IL1beta, and actin filaments visualized by staining with green-labeled phalloidin (a), and MUC1-ARF protein immunostained with anti MUC1-ARF MPR2G10 followed by detection with red-labeled secondary antibody (b). DAPI-stained nuclei and merged images are shown in (c) and (d), respectively. Following prolonged cytokine treatment, MUC1-ARF expression shifted in part to membrane protrusions, indicated by yellow arrows. (E) Cell lysates were prepared from HK293 cells transiently transfected with an expression vector driving expression of MUC1 cDNA (lanes 2) or with a control empty expression vector (lanes 1). Anti-MUC1-ARF immunprecipitates were resolved on SDS-PAGE, western blotted, and probed with antibodies as indicated.

Cytokines such as interferon-gamma, tumor necrosis factor-alpha (TNF), IL-1beta and IL-6, and combinations of these cytokines, have been shown to elicit marked increases in MUC1-TM expression [26, 32–38]. Although interferon-gamma by itself was shown to upregulate MUC1 expression in most cell lines, the addition of tumor necrosis factor-alpha to interferon-gamma elicited marked synergistic stimulation of MUC1 expression [26, 33]. Specifically, combinations of the cytokines [interferon-gamma and IL-1beta], [IL-6 together with TNFalpha] and [interferon-gamma and TNFalpha] have been shown to stimulate MUC1-TM expression to several-fold higher than by each cytokine alone [26]. We therefore looked at the effect of these combinations of cytokines on MUC1-ARF expression. Similarly to cytokine stimulation of MUC1-TM expression, MUC1-ARF showed both quantitative and qualitative changes following cytokine treatment (Fig 10). Immunofluorescence with mAb MPR2G10, combinations of interferon-gamma and IL-1beta, IL-6 together with TNFalpha, and of interferon-gamma and TNFalpha clearly led to increased MUC1-ARF expression (Fig 10A, compare Panels ‘b’, 'c' and 'd', with Panel 'a'). Flow cytometry analyses and quantitative ELISA assays using anti-MUC1-ARF monoclonal antibody MPR2G10 confirmed these findings (Fig 10B, Panels 'a', 'b' and 'c').

As expected, prolonged cytokine treatment with interferon-gamma and IL-1beta clearly induced MUC1-TM. Irrespective of cytokine addition, MUC1-TM remained tethered at the cell membrane (Fig 10C, compare panels a and b). In contrast, prolonged cytokine treatment elicited a disproportionate increase in cytoplasmic MUC1-ARF at the expense of nuclear MUC1-ARF (Fig 10C, compare panels a' and b'), indicating that exposure to the cytokines leads not only to a quantitative increase in MUC1-ARF but also to cellular relocalization of MUC1-ARF. This shift was particularly prominent following extended cytokine treatment, and discrete compartments that stained intensely for MUC1-ARF appeared to be in the process of forming blebs at the cell surface (Fig 10D, indicated by yellow arrows).

### MUC1-ARF function: The proline-rich tandem repeat domain of MUC1-ARF interacts with SH3-domain containing signaling proteins

In an attempt to define MUC1-ARF function, we used a bioinformatic MotifScan approach to search for proteins putatively interacting with MUC1-ARF. This yielded a set of signaling proteins such as Grb2, phospholipase C-gamma1, p85 subunit of PI-3'-kinase, and Abl tyrosine kinase which were predicted to bind via their SH3 domains with a high level of confidence (>99.9^th^ percentile) to proline-rich regions of MUC1-ARF tandem repeat sequence GRPRAPP**P**PQPTVSP. To see whether MUC1-ARF in fact forms complexes with SH3-containing proteins as predicted, anti-MUC1-ARF immunoprecipitates (IP) were resolved by SDS-PAGE and probed by immunoblotting (IB) with an array of antibodies recognizing SH3–domain containing proteins. These assays demonstrated that in fact Abl tyrosine kinase and cortactin, both of which contain SH3 domains co-precipitated with MUC1-ARF from MUC1 cDNA transfected HK cells (Fig 10E, lanes 2, panels 'a' and 'b'). HK cells transfected with mock cDNA and therefore not expressing endogenous MUC1 did not show the coprecipitating proteins (Fig 10E, lanes 1).

To further our understanding of MUC1-ARF function, it would be highly informative to knockout MUC1-ARF expression in cells that naturally express MUC1-ARF protein and look at the resulting phenotype. However the entire MUC1-ARF protein coding region, including the initiation codons directing MUC1-ARF protein expression, is an integral component of the MUC1-TM reading frame (Fig 2). As a result, knocking out MUC1-ARF expression inevitably means that MUC1-TM protein produced in these cells will be truncated. Whatever phenotype is seen in such cells could not be unambiguously ascribed to the lack of MUC1-ARF; it could just as well be attributed to expression of a truncated MUC1-TM protein. Thus knocking out MUC1-ARF expression, for example by CRISPR technology, is not a feasible experimental approach to learn about MUC1-ARF function.

Much information on MUC1-TM function has been gained by identifying proteins that interact with MUC1-TM. In a similar manner, identification of proteins interacting with MUC1-ARF in cells that natively express MUC1-ARF could shed light on MUC1-ARF function. To identify such proteins, we prepared lysates from MCF7 cells (that express MUC1-ARF, see above Fig 6) and MUC1-ARF protein was immunoprecipitated using anti-MUC1-ARF monoclonal antibody MPR2G10 prebound to Protein A/G agarose beads. Proteins associated with the beads were then trypsin digested, followed by mass spectrometric analyses. Addition of MUC1-ARF repeat peptide consistently competed out MPR2G10 monoclonal antibody immunoreactivity in all modalities employed including immunofluorescence, immunohistochemistry, ELISA assays and western blotting, and in all cells expressing MUC1-ARF including cell transfectants expressing the human *MUC1* gene, cell lines endogenously expressing the *MUC1* gene, and in human tissues expressing the *MUC1* gene. To eliminate the possibility of non-specific antibody binding, MUC1-ARF protein was immunoprecipitated, either in the absence of competing MUC1-ARF peptide or in its presence. Only those proteins that showed a significant mass spectrometric signal in the absence of ARF peptide and a complete 'competing-out' of signal (a reading of "0") in the presence of ARF peptide were deemed to be true proteins that interact with MUC1-ARF. This analysis yielded two prominently interacting proteins, Glucose-6-phosphate dehydrogenase (G6PD) and Dynamin 2 (DNM2), whereas all other proteins gave substantially lower signals. Tryptic digestion releases Protein A and Protein G peptides from the affinity support matrix (Protein A/G agarose beads)- these peptides represent approximately 1.5% of total spectra. As an indication of their abundance, G6PD and DNM2 yielded percentage values of total spectra of 0.16% and 0.11%, respectively, and both proteins gave zero readings in the samples that had been immunoprecipitated in the presence of MUC1-ARF peptide demonstrating their binding specificity. When viewed in relation to the abundant Protein A and Protein G peptides, the values recorded for G6PD and DNM2 are exceptionally high.

## Discussion

We demonstrate here that translation of MUC1 mRNA in an alternate reading frame generates MUC1-ARF protein, which differs in its entirety from the amino acid sequence of its tumor-associated 'parent' protein, MUC1-TM (Figs 1 and 2). MUC1-ARF is comprised of 49 amino acids N-terminal to the tandem repeat domain, followed by the tandem repeat domain and finally by a C-terminal domain of 175 amino acids. Since the MUC1-TM tandem repeat domain has a lower limit of about 300 amino acids (15 copies of a twenty amino acid repeat unit [19, 25, 39–41]), the complete MUC1-ARF protein must contain at least 524 amino acids making it as far as we are aware, the largest protein reported to date generated by an overlapping alternate reading frame.

The studies reported here demonstrate unequivocal expression of the frameshifted MUC1-ARF protein in MUC1 cell transfectants, in MUC1-expressing cancer cell lines, and in sections of normal and malignant human tissues. In normal tissues, expression of the MUC1-ARF and MUC1-TM proteins showed a tight correlation, such that epithelial cells forming ducts in the pancreas and kidney expressed not only MUC1-TM, but also clearly expressed MUC1-ARF (Figs 7 and 8). The contrary was also true: tissues such as brain and liver which do not express MUC1-TM do not express MUC1-ARF. Similarly, ductal epithelial cells of resting breast tissue showed very low MUC1-TM expression, accompanied by almost undetectable levels of MUC1-ARF.

Mass spectrometric analyses were ineffective in identifying peptides contained within the MUC1-ARF protein, and several possibilities could explain the inability of this modality to identify MUC1-ARF. It may well be that MUC1-ARF undergoes post-translational modifications on multiple amino acid residues (see above in Results for the MotifScan analysis), the type of which we do not presently know, rendering its identification by mass spectrometry intractable. Such post-translational modifications or combinations thereof appearing in the protein either stoichiometrically or/and substoichiometrically, could include, yet not be restricted to, methylation, phosphorylation and/or O-GlcNAcylation [42], the latter of which is known to be particularly prevalent on nuclear-resident proteins, such as MUC1-ARF. In this context it is pertinent to note that the latest review of a flurry of recent studies uncovering the unexplored alternative ORF proteome (AltORFs) [43], notes that mass spectrometry experiments often lack the sensitivity or technical details required for detection of all proteins, as appears to be the case for MUC1-ARF.

In contrast to this, the evidence presented here that MUC1-ARF is expressed as a protein is strikingly convincing: (i) its expression is seen only in cells that express the human *MUC1* gene (Figs 3, 4, 6, 7 and 8) (ii) mouse cells that do not express human MUC1 are negative for MUC1-ARF whereas the same cells transfected with and expressing human MUC1 DNA express MUC1-ARF, as observed by immunofluorescence, western blots, flow cytometry and ELISA assays (Figs 3 and 4), (iii) MUC1-ARF expression was detected not only with the anti-MUC1-ARF monoclonal antibody MPR2G10, but also with purified anti-MUC1-ARF polyclonal antisera as well as with an additional two independently isolated anti-MUC1-ARF monoclonal antibodies (Fig 3), and (iv) MUC1-ARF immunoreactivity was in all cases abrogated by the addition of MUC1-ARF specific peptide, and not by non-specific peptides. Constituting perhaps the most forceful piece of evidence, (v) the immunohistochemical analyses performed on unmanipulated normal human tissues clearly show MUC1-ARF protein that localizes to the cell nucleus (Figs 7, 8 and 9). Particularly compelling is the picture seen with normal kidney tissue (Fig 7) where MUC1-ARF localizes to the nuclei of only those epithelial cells lining the ducts of distal tubules. All other cells within the same section of kidney tissue are unequivocally negative for MUC1-ARF. In the kidney, this discrete expression of MUC1-ARF correlates precisely with the distribution of MUC1-TM expression, which is restricted solely to the identical ductal-forming epithelial cells of the distal tubules. Comparison of sequential kidney sections stained for either MUC1-TM or MUC1-ARF clearly reveals that those cells expressing MUC1-TM also express MUC1-ARF. Within the same section, epithelial cells other than those forming the lumen of distal tubules serve admirably both for antibody specificity and as negative controls. They are all negative not only for MUC1-TM but also for MUC1-ARF. Further bolstering this evidence for the in-vivo expression of MUC1-ARF are the analyses of MUC1 protein expression in the normal pancreas (Figs 7 and 8). In the pancreas, a similar situation pertains to that seen in the kidney, in that both MUC1-TM and MUC1-ARF are clearly expressed in acinar and ductal cells of the exocrine pancreas (Figs 7 and 8). In contrast to the pancreatic acinar and ductal cells, the pancreatic islets forming the endocrine pancreas do not express MUC1-TM as we demonstrate here (Fig 8) and as is known from the literature [28]. Consistent with this, MUC1-ARF is unambiguously absent from the pancreatic islet cells, just as is MUC1-TM (Fig 8).

Additional evidence for expression of MUC1-ARF protein was provided by (vi) Analyses of MUC1-ARF protein expression by western blots in cell lines that were in parallel analyzed for expression of MUC1 mRNA by qPCR analyses (Fig 6). Without exception, we observed an absolute correlation of MUC1-ARF protein expression to expression of MUC1 mRNA. The converse was also true in that all cell lines that did not show MUC1 mRNA expression, were likewise negative for MUC1-ARF protein (Fig 6). (vii) Polymorphic allelic forms of the MUC1-ARF protein were observed in those cell lines expressing the MUC1-ARF protein, consistent with the known allelic polymorphism of the *MUC1* gene itself (Fig 6). Finally, (viii) the smaller MUC1-ARF allele was always expressed at significantly higher levels as compared to expression of the larger allele consistent with previously published data on expression of MUC1 mRNA (25). Taken together, the experimental evidence detailed in the Results section (Figs 1, 3–10), and summarized above [items (i)–(viii)] compellingly favors MUC1-ARF protein expression and makes it highly unlikely, if not untenable, that the MUC1-ARF protein is an experimental artifact.

Among its multiple functions, cell-surface MUC1-TM acts to protect the cell from foreign organisms such as bacteria and viruses [44–47]. Consistent with this is our finding that *MUC1* gene expression, including that of MUC1-ARF, is markedly upregulated by interferons and other cytokines (Fig 10). Correspondingly, a single STAT responsive element has been identified within the MUC1 promoter, that when mutated both decreases MUC1 promoter activity in breast cancer cells and also abolishes stimulation by interleukin-6 and interferon-gamma [35]. Expression of both MUC1-TM and MUC1-ARF may in fact be favored in environments that are conducive to the growth of foreign organisms, such as within the lumen of distal renal tubules, as we observed here (Fig 6). In keeping with this, resting normal breast comprising ductal tissue through which little if any fluid flows demonstrates low expression of both MUC1-TM and MUC1-ARF. This contrasts with lactating breast, where high levels of MUC1-TM protein are present on the apical surfaces of epithelial cells forming ducts involved in lactation [48].

It has been amply documented that MUC1-TM transduces signals from the extracellular space into the cell via tyrosine phosphorylation of its cytoplasmic domain [49] which then recruits second messenger signaling proteins such as Grb2 and beta catenin [49–54]. Following its binding to STAT3 and nuclear factor-kappa-b (NF-kappa-b), the cytoplasmic MUC1-TM domain re-localizes from the cell membrane to the cell nucleus, and can modulate the activity of the NF-kappa-b pathway by interacting with, and activating NF-kappa-b p65 and family members of IKK [55]. It is intriguing therefore that MUC1-ARF not only localizes primarily to the cell nucleus, but also binds the SH3-domain containing proteins Abl and cortactin. In fact, because of the tandem repeat of its proline-rich binding motif, MUC1-ARF protein likely contains multiple docking sites for signaling proteins, suggesting that MUC1-ARF may serve as a scaffold capable of binding multiple copies of [SH3-domain]-containing proteins. It is notable then that the cytoplasmic domain in the beta-subunit of the MUC1-TM protein undergoes phosphorylation on a series of tyrosine residues thereby complexing to SH2-domain-containing signaling proteins including the Abl tyrosine kinase [56]. Furthermore, like the Abl tyrosine kinase [57] MUC1-ARF localizes both to the cytoplasm and to the nucleus, suggesting that MUC1-ARF could serve to facilitate the shuttling of bound signaling proteins to and from the nucleus.

Following the initial discovery of MUC1-TM tyrosine phosphorylation [49] understanding MUC1-TM function has been considerably enhanced by identifying proteins interacting with the MUC1-TM protein. A similar approach was therefore used here to gain insight into MUC1-ARF function. By mass spectrometric analyses of proteins interacting with MUC1-ARF, we found that Glucose-6-phosphate 1-dehydrogenase (G6PD) and Dynamin 2 (DNM2) were the highest scoring proteins interacting with MUC1-ARF. Recent work has demonstrated that MUC1-TM acts as a novel metabolic master regulator by acting as a transcriptional coactivator thereby regulating expression of metabolic genes [reviewed in [58]]. Because of this, the interaction of MUC1-ARF with G6PD, an important metabolic regulator, is particularly intriguing. G6PD is a significant metabolic regulator that provides reducing power (NADPH) and pentose phosphates for fatty acid and nucleic acid synthesis. MUC1-ARF interaction with G6PD may act to modulate G6PD activity thereby acting similarly to MUC1-TM as a metabolic regulator (58).

MUC1-ARF interaction with dynamin 2, a GTPase that facilitates vesicle fission during synaptic vesicle endocytosis, is also of interest because the C-terminal domain of dynamin is a proline-rich domain (PRD) very similar in make-up to the proline-rich domain of the MUC1-ARF tandem repeats [59, 60]. Dynamin is recruited to sites of endocytosis by interacting with the SH3 domains of a variety of proteins, via dynamin PXXP motifs (where X represents any amino acid) that are flanked on either side by a basic residue such as arginine. Such PXXP motifs are particularly prevalent in the MUC1-ARF tandem repeats (see Results), and appear there as PRAP, PPPP, PPQP, PRTP and PGRP. It therefore may well be that protein(s) containing two or more SH3 domains bridge between dynamin 2 and MUC1-ARF. Significantly, dynamin interacts with cortactin, which comprises a well characterized SH3 domain [61, 62] and is an actin-binding protein shown to participate in receptor-mediated endocytosis. Regulation of receptor-mediated endocytosis requires remodeling of actin filaments involving dynamin2 GTPase activity that is dependent on its interaction with the F-actin-binding protein cortactin [63]. Because it has been extensively documented that cell surface receptor regulation involves both dynamin and a number of SH3 domain-containing proteins such as cortactin (for example [64–67], the interaction of MUC1-ARF not only with dynamin 2 but also with cortactin (Fig 10, Panel E) suggests that MUC1-ARF may also be involved in the regulation of cell surface receptors. As compelling as the above evidence is, the definitive nature of MUC1-ARF function remains to be determined.

Parental and ARF proteins expressed from the same gene are often functionally linked. For example, the proteins p16INK4A and p19ARF proteins are both expressed from a common ARF/INK4A locus, and act as tumor suppressor proteins and function in similar pathways [8]. Interestingly, the protein ALEX derived by alternate frame reading of the mRNA coding for the G-protein alpha-subunit XLas physically interacts with the XL-domain of the ‘parent’ XLas gene product, such that both parent and ARF proteins act in concert [10, 68]. Such physical interaction between 'parent' and ARF proteins is also seen between Xbp1(S) protein, a transcription factor that participates in the unfolded protein response, and Xbp1(U)-ARF protein generated by alternate reading frame translation of an unspliced Xbp mRNA [69, 70]. Similarly, the overlapping reading frame in the ataxin-1 coding sequence encodes a novel protein that also interacts with the 'parental' ataxin-1 protein [71]. The pattern emerging from these findings is that despite very different amino acid sequences of expressed ARF proteins compared to their 'parent' proteins, ARF proteins can be functionally linked to their 'parent' proteins. We do not as yet know with any degree of certainty, whether the MUC1-TM and MUC1-ARF proteins are in fact functionally linked as is the case for parental and ARF proteins cited above.

MUC1-ARF is a very basic protein, rich in proline residues that comprise about 35% of the tandem repeat sequence. This structural make-up is in line with other ARF proteins that are basic and show a conspicuous bias for a high proline content suggesting an increase in the level of structural disorder. For example, the ALEX protein has a predicted pI of 11.8 with a 21% proline content [10] and the ARF protein derived from the INK4 locus is composed of approximately 20% arginine residues [8]. Because of its high proline content, MUC1-ARF appears to be natively unstructured, just as are ALEX and p19^ARF^. Of note, both ALEX and p19^ARF^ form specific and tight interactions with partner proteins, and p19^ARF^ acquires activity and stability only when bound to targets [72].

In contrast to localization of MUC1-TM to the cell surface, MUC1-ARF localizes to the cell nucleus, as observed in normal kidney and pancreas as well as in breast cancer tissues (Figs 5–8). The cytoplasmic domain of MUC1-TM has been observed to also localize to the cell nucleus [50], thus highlighting the similarity of MUC1-ARF and MUC1-TM.

Proteins locating at and functioning in the nucleus frequently contain nuclear localization signals (NLS), required for translocation through the nuclear pore complex. These typically contain clusters of basic amino acids, usually flanked by proline residues, and many NLS motifs appear in pairs (bipartite NLS signals) or even as multiple signals within the one protein [73]. Although there is no canonical nuclear localization signal present in the MUC1-ARF protein, it is notable that the MUC1-ARF tandem repeat itself comprises the proline-arginine rich motifs *PRAPP*, *PRPR* and *GRPR*. In this context, various FGF2 and FGF3 protein isoforms, especially those initiating at upstream alternative initiation sites, localize to the cell nucleus [74–76] not because they contain a canonical nuclear localization signal, but because they also comprise motifs such as PRAAP, PRTR and GRGR very similar to those seen in the MUC1-ARF tandem repeat (compare FGF/*MUC1-ARF* motifs- PRAAP*/PRAPP*, PRTR/*PRPR* and GRGR/ *GRPR*). It is therefore possible that just as in the case of the FGF2 and FGF3 proteins, proline-arginine rich motifs within the MUC1-ARF tandem repeat direct nuclear localization of MUC1-ARF.

In contrast to the significant expression of both MUC1-TM and of MUC1-ARF on the apical surfaces of normal luminal epithelial cells present both in the kidney and those forming the exocrine pancreatic ducts and acini (Figs 7 and 8), the normal luminal epithelial cell of the resting non-lactating breast expresses very low levels if at all of these MUC1 proteins. Epithelial cells of breast cancer tissue, however, show in many instances very high MUC1-TM expression. The accepted consensus is that this increased expression in breast cancer cells in some way links MUC1-TM protein to a malignant phenotype [see review [15]].

Increased expression of MUC1-TM taken as the single parameter, however, without taking into account the tissue architecture of MUC1-expressing cells has been shown not to have prognostic significance [77]. In a study encompassing more than 1,300 cases of breast cancer it was clearly shown that the specific tissue architecture of MUC1-expressing cells has prognostic value: cytoplasmic expression with *circumferential* membranous MUC1-TM localization was unmistakably associated with a worse prognosis, whereas apical, luminal expression predicted a favorable outcome. Thus not only is tissue type (breast tissue versus kidney and/or pancreatic tissues) important in considering the relevance of MUC1 expression to malignant phenotype, but also the spatial organization of MUC1 expression on malignant epithelial cells can be determinant. Breast cancer cannot be considered to be one homogeneous disease. Indeed molecular expression signatures [78] have identified at least 5 major breast cancer types- luminal A, luminal B, basal, HER2-enriched and normal-like [[79] and references contained therein], and although this broad classification has prognostic implications, such prognostic forecasting is not at all times clear-cut. The luminal A subtype, with a better prognosis, and luminal B subtype with a worse prognosis are both considered to be derived from the breast luminal epithelial cell. Yet these two subtypes are particularly difficult to differentiate one from the other, and in this regard our studies on MUC1-ARF expression may help to discriminate the luminal A and luminal B subtypes. MUC1-ARF expression, as shown here, is seen only in a select sub-group (~40%) of those breast tumors that express MUC1-TM, and thus may designate a breast cancer subtype with a specific prognostic outcome for the following reasons. Because *normal* pancreatic and kidney cells coexpress both MUC1-TM and MUC1-ARF, thus typifying the MUC1 expression pattern of a normal epithelial cell (both MUC1-TM and MUC1-ARF), it would appear as if the subtype of breast cancers that also coexpress both MUC1-TM and MUC1-ARF, a signature MUC1-expression pattern reflecting the normal epithelial cell, may have a better prognosis than those tumors that express only MUC1-TM. The tissue architecture of MUC1-TM and MUC1-ARF expressing cells, as described above, might also be a factor. The unambiguous prognostic significance of MUC1-ARF expression (or the lack of its expression) together with MUC1-TM will require the further analysis of much larger population samples.

In cancer tissues, expression of MUC1-ARF protein was highly dependent on the tissue in question. Pancreatic cancers, known for their aggressive malignant phenotype, showed a loss of MUC1-ARF expression, as compared to high MUC1-ARF expression in epithelial cells from normal pancreatic tissue. In contrast, a number of human breast cancer samples showed preferential MUC1-ARF expression in the cancer cell population, with little to no expression in normal breast tissue, suggesting that just as the transmembrane MUC1 protein is preferentially overexpressed in transformed breast epithelial cells, so too is the MUC1-ARF protein. Although in these samples MUC1-ARF expression segregated with MUC1-TM expression, it did so in only a restricted subset of the MUC1-TM positive samples, implying that mechanisms other than the mere presence of MUC1 mRNA determine MUC1-ARF expression and that regulatory controls ultimately govern which reading frame(s) are translated, and to what extent. Possible mechanisms include leaky scanning, ribosomal shunting and mechanisms promoting use of internal ribosomal entry sites. As demonstrated here, a diversity of cytokines induce MUC1-ARF expression, suggesting that a specific milieu of cytokines and growth factors present in the tumor microenvironment may govern its restricted expression. This in turn could define a cancer subtype, wherein MUC1-ARF positivity confers prognostic significance, as also discussed above. The intracellular distribution of MUC1-ARF protein, whether nuclear or/and cytoplasmic, may also be important, and extensive immunohistochemical studies will be required to see whether the quantitative and qualitative changes in MUC1-ARF expression in breast cancer tissue indeed have prognostic significance to disease outcome.

The expression of a given protein by normal non-malignant cells does not preclude linkage of that protein to a malignant phenotype. This linkage to malignancy may be seen as an altered pattern of expression of the protein, that could involve a quantitative alteration in its level of expression or/and in its cellular localization. A case in point, clearly pertinent to this report is that of MUC1-TM itself, which is universally accepted as linked in some way to breast cancer (see above and for a recent review [15]), despite the fact that MUC1-TM is extensively expressed on epithelial cells of, amongst others, the normal pancreas, kidney and normal, lactating breast. Further emphasizing this point are the well-known examples of the normal cellular proteins epidermal growth factor receptor (EGFR), HER2 (epidermal growth factor receptor 2) and estrogen receptor (ER). It is their *inappropriate* expression that inextricably links these proteins to a malignant phenotype, and it is universally accepted that EGFR, HER2 and ER are unambiguously linked to the breast cancer cell. Indeed cancer therapies such as antibodies targeting EGFR and HER2 [for example [80, 81]] as well as inhibitors of estrogen receptor activity are extensively used in the clinic. Yet notwithstanding their undisputed relevance to cancer, it is a well-known fact that EGFR, HER2 and ER are all expressed in normal cells, in which they play critical roles in normal cell growth and differentiation [82]. It is their inappropriate expression- quantitatively and qualitatively, spatially and temporally- just as we show here for MUC1-ARF, that links these proteins to the malignant cell.

The murine *MUC1* gene and its protein product [83] show major differences compared to those of primates and humans. In the mouse, the region spanning from the MUC1-TM initiation AUG codon to the tandem repeat domain encompasses only 41 amino acids, whereas in humans it comprises 125 amino acids. It is precisely within this segment that the human ARF initiation codon is located and it is likely that because of this difference between human and mouse, the mouse *MUC1* gene does not generate a MUC1-ARF protein. The mouse tandem repeat domain contains two additional crucial differences from that of primates: Whereas in primates the number of repeats can vary in any allele from ~15–125 repeats [19], the number of repeats is fixed in the mouse at an invariable 16 repeats [83]. Furthermore, the mouse repeat sequences themselves are far more divergent from each other as compared to the human and primate repeat sequences which are very similar one to the other [25, 84]. It thus appears that mammals higher up on the evolutionary ladder, such as primates and humans, have developed a more complex *MUC1* gene that is reflected in differences in the region from the MUC1-TM start codon until the tandem repeat domain as well as in the repeat domain itself. Although facilitating the synthesis of the MUC1-ARF protein in addition to MUC1-TM as shown here, this sophistication exacts a price from the cellular machineries that must cope with the non-trivial tasks of faithful replication and transcription of highly conserved GC-rich sequences, as found in the tandem repeat sequences and those immediately flanking it. That mistakes can occur here is amply demonstrated by the recent report on a mutation within the MUC1 tandem repeat which results in medullary cystic kidney disease 1 (MCKD1)[85].

The human *MUC1* gene has been previously shown to generate not only the transmembrane mucin-like protein that comprises a highly glycosylated tandem repeat domain, but additional alternative isoforms designated MUC1-X, MUC1-Y and MUC1-ZD [20–22, 86], that are generated by differential splicing events. Like MUC1-TM, both MUC1-X and MUC1-Y are transmembrane proteins. Yet in distinction to MUC1-TM they are both devoid of the central tandem repeat domain, that is spliced out using splice donor and acceptor sites located upstream and downstream to the tandem repeat array. Interestingly, the MUC1-ZD protein [86] utilizes the same splice donor site to that used by MUC1-X and MUC1-Y, yet the alternative MUC1-ZD splice acceptor site located downstream to the tandem repeat array leads to a downstream frameshifted sequence initiating with the amino acid sequence IPAPTTTKSCR… and terminating with GQDLWWYN. This C-terminal region of MUC1-ZD contains 43 amino acids, a region that is identical to the C-terminal portion of the MUC1-ARF protein reported here. Despite the identity of this C-terminal region, it is clear that these two MUC1 proteins localize to different cellular/extracellular compartments- MUC1-ZD is likely a secreted protein [86], whereas MUC1-ARF is a nuclear and cytoplasmic protein, as shown here. The overall scheme, that of a secreted protein and a nuclear/cytoplasmic protein both being derived from the same gene, is also observed with the FGF proteins. Some FGF protein isoforms are secreted proteins and others localize to the cytoplasmic/nuclear compartments, yet all derive from the same gene [for example [87, 88]].

Until very recently the scope of proteome diversity in eukaryotic organisms was thought to be determined primarily by canonical mechanisms such as utilization of alternative promoters, alternative splicing, usage of alternative adenylation sites and RNA editing. This canon is now being seriously challenged by the realization that enhancement of proteome diversity by non-canonical mechanisms, such as dual-coding mRNAs [43], wherein a single mRNA is decoded not only in the canonical reading frame, but also in alternative reading frames, is far more common than previously envisioned [2]. Here we have shown that the *MUC1* gene generates a MUC1 mRNA that yields more than a single protein, resulting not only in the well-characterized tumor-associated MUC1-TM protein, but also a novel MUC1-ARF protein. Furthermore MUC1-ARF represents the longest eukaryotic ARF protein heretofore reported. In addition to enhancing our understanding of *MUC1* gene involvement in the malignant transformation of cells, the discovery of MUC1-ARF furthers, at the more general level, our appreciation of dual-coding mechanisms in expanding proteome diversity.
